# Effectiveness of building-level sewage surveillance during both community-spread and sporadic-infection phases of SARS-CoV-2 in a university campus population

**DOI:** 10.1093/femsmc/xtac024

**Published:** 2022-09-24

**Authors:** William Johnson, Katelyn Reeves, Jennifer Liebig, Antonio Feula, Claire Butler, Michaela Alkire, Samiha Singh, Shelby Litton, Kerry O'Conor, Keaton Jones, Nikolas Ortega, Trace Shimek, Julia Witteman, Sampling Team Collaborators, Elle Coe, Heidi Heuer, Jeffrey Jones, Sara Key, Jacob Lilienfeld, Juniper Maggi, Lauren Nelson, Kevin Pulley, Paul Wilkerson, Bailey Vigil, Gordon Zak, Kiersten Maxwell, Madeline Karr, Nicholas Freeman, Emily Saldana, Lewis Salveson, Kate Tomlinson, Jorge Vargas-barriga, Kristen K Bjorkman, Cresten Mansfeldt

**Affiliations:** Department of Civil, Environmental, and Architectural Engineering, University of Colorado Boulder, 1111 Engineering Drive, Boulder, CO 80309, United States; Environmental Engineering Program, University of Colorado Boulder, 4001 Discovery Drive, Boulder, CO 80303, United States; Department of Civil, Environmental, and Architectural Engineering, University of Colorado Boulder, 1111 Engineering Drive, Boulder, CO 80309, United States; Environmental Engineering Program, University of Colorado Boulder, 4001 Discovery Drive, Boulder, CO 80303, United States; BioFrontiers Institute, University of Colorado Boulder, 3415 Colorado Avenue, Boulder, CO 80303, United States; BioFrontiers Institute, University of Colorado Boulder, 3415 Colorado Avenue, Boulder, CO 80303, United States; Environmental Engineering Program, University of Colorado Boulder, 4001 Discovery Drive, Boulder, CO 80303, United States; Environmental Engineering Program, University of Colorado Boulder, 4001 Discovery Drive, Boulder, CO 80303, United States; Environmental Engineering Program, University of Colorado Boulder, 4001 Discovery Drive, Boulder, CO 80303, United States; Environmental Engineering Program, University of Colorado Boulder, 4001 Discovery Drive, Boulder, CO 80303, United States; Environmental Engineering Program, University of Colorado Boulder, 4001 Discovery Drive, Boulder, CO 80303, United States; Environmental Engineering Program, University of Colorado Boulder, 4001 Discovery Drive, Boulder, CO 80303, United States; Environmental Engineering Program, University of Colorado Boulder, 4001 Discovery Drive, Boulder, CO 80303, United States; Environmental Engineering Program, University of Colorado Boulder, 4001 Discovery Drive, Boulder, CO 80303, United States; Environmental Engineering Program, University of Colorado Boulder, 4001 Discovery Drive, Boulder, CO 80303, United States; BioFrontiers Institute, University of Colorado Boulder, 3415 Colorado Avenue, Boulder, CO 80303, United States; Department of Civil, Environmental, and Architectural Engineering, University of Colorado Boulder, 1111 Engineering Drive, Boulder, CO 80309, United States; Environmental Engineering Program, University of Colorado Boulder, 4001 Discovery Drive, Boulder, CO 80303, United States

**Keywords:** wastewater-based epidemiology, building-level, SARS-CoV-2, norovirus, influenza

## Abstract

Pathogen surveillance within wastewater rapidly progressed during the SARS-CoV-2 pandemic and informed public health management. In addition to the successful monitoring of entire sewer catchment basins at the treatment facility scale, subcatchment or building-level monitoring enabled targeted support of resource deployment. However, optimizing the temporal and spatial resolution of these monitoring programs remains complex due to population dynamics and within-sewer physical, chemical, and biological processes. To address these limitations, this study explores the advancement of the building-scale network that monitored the on-campus residential population at the University of Colorado Boulder between August 2020 and May 2021 through a daily SARS-CoV-2 surveillance campaign. During the study period, SARS-CoV-2 infection prevalence transitioned from robust community spread in Fall 2020 to sporadic infections in Spring 2021. Temporally, these distinct phases enabled investigating the effectiveness of resource commitment by exploring subsets of the original daily sampling data. Spatially, select sampling sites were installed along the flow path of the pipe network, enabling the exploration of the conservation of viral concentrations within the wastewater. Infection prevalence and resource commitment for informed action displayed an inverted relationship: higher temporal and spatial resolution surveillance is more imperative during sporadic infection phases than during high prevalence periods. This relationship was reinforced when norovirus (two minor clusters) and influenza (primarily absent) were additionally surveilled at a weekly frequency. Overall, resource commitment should scale to meet the objectives of the monitoring campaign—providing a general prevalence estimate requires fewer resources than an early-warning and targeted-action monitoring framework.

## Teaser

The resource commitment required for wastewater-based surveillance inversely scales with infection prevalence.

## Introduction

Wastewater-based surveillance (WBS) has been successfully applied to monitor entire sewage–drainage catchments to individual buildings as a public health response to the coronavirus disease (COVID-19) pandemic (Keshaviah et al. 2021, Kirby et al. 2021, McClary-Gutierrez et al. 2021, Naughton et al. 2021). The second emergent severe acute respiratory syndrome coronavirus (SARS-CoV-2), which causes COVID-19 is responsible for an estimated 594,000,000 infections and 6,446,000 deaths as of 22 August 2022 (World Health Organization 2019, Medema et al. 2020, Peccia et al. 2020, Spurbeck et al. 2021). Globally, select college campuses adopted WBS to monitor on-campus residences and complement individualized medical and screening programs (Barich and Slonczekski 2021, Betancourt et al. 2021, Bivins and Bibby 2021, Bivins et al. 2021, Brooks et al. 2021, Corchis-Scott et al. 2021, Fahrenfeld et al. 2021, Gibas et al. 2021, Karthikeyan et al. 2021, Landstrom et al. 2022, Liu et al. 2021, Reeves et al. 2021, Scott et al. 2021, Sharkey et al. 2021, Sweetapple et al. 2021, Tang et al. 2022, Vo et al. 2022, Wang et al. 2022, Wright et al. 2022). As of 22 August 2022, 282 universities are publicly reported to have run WBS campaigns targeting SARS-CoV-2 (Naughton et al. 2021). However, the total population covered by these campaigns remains ambiguous resulting from the combined factors of incomplete or transient monitoring of those on campuses (e.g. inability to monitor specific locations, commuters, and employees). These ambiguities amplify when applying WBS within sewage conveyance networks beyond university campus settings, raising a cost-benefit question surrounding the resource commitment required to achieve a public health impact when designing and operating these campaigns (Peccia et al. 2020, Weidhaas et al. 2021).

The main intent of monitoring smaller sewer catchments or at the building-level is being able to deploy additional individualized testing in response to results from wastewater, identify infected individuals rapidly, and thus medically intervene early to prevent severe disease and further viral spread (Betancourt et al. 2021, Fahrenfeld et al. 2021). However, the sampling frequency (often ranging from daily to weekly in previous publications) influences the effectiveness of this approach (Haak et al. 2022). Additionally, the number of sampling locations targeting a specific community determines the spatial resolution of and required analytical sensitivity needed by the WBS campaign. Optimizing this spatial deployment requires a better understanding of the alteration in the targeted signal along the sewer network and the development of, and standardization to, effective processing controls (Ahmed et al. 2020, Kantor et al. 2021, LaTurner et al. 2021, McClary-Gutierrez et al. 2021, McCall et al. 2022, Xie et al. 2022). Alterations in social activity also influence the prevalence of SARS-CoV-2, and exploring the dynamics captured by WBS data or overlaying the results onto other human behavior datasets may inform the spatial and/or temporal resource commitment required to monitor a given community effectively (Wright et al. 2022). These social interactions may propagate not only SARS-CoV-2 infections but also a wide range of other pathogens. Prior to deploying WBS for these new targets, each virus requires additional validation and optimization. For example, norovirus is a commonly identified pathogen shed within feces, whereas influenza prevalence has been previously shown to be disconnected from wastewater concentrations, both providing unique considerations for WBS (Grøndahl-Rosado et al. 2014, Kitajima et al. 2010, Katayama et al. 2008, Nordgren et al. 2009, Heijnen and Medema 2011).

To address these points, the high-resolution WBS dataset collected at the University of Colorado Boulder (CU Boulder) was reconsidered to explore the needed spatial and temporal resource commitment. The daily on-campus sewage collection campaign at CU Boulder operated from the Fall 2020 (August–November 2020) into the Spring 2021 (February–May 2021) semester in concert with campus individualized diagnostic and screening programs (Reeves et al. 2021). With the daily nature of the samples being collected across 23 sites, this study compares subsets of the data representing different sampling frequencies (e.g., every other day, weekly) to determine the effectiveness at capturing and reflecting clinically detected infections. This study also utilizes the high number of sites to explore the conservation of detected signals along pipe networks to examine the appropriateness of sampling locations. Finally, this study expands into developing and deploying tools to better standardize the data using the pepper mild mottle virus (PMMoV) and to monitor the on-campus spread of influenza and norovirus, adapting WBS into a more comprehensive tool for community health monitoring.

## Materials and methods

### Sample collection and SARS-CoV-2 processing

The Fall methods and data, spanning 25 August 2020–23 November 2020, was previously presented (Reeves et al. 2021). Briefly, 24-hour composite samples were withdrawn from 23 distinct wastewater flows using constructed autosamplers at manholes around the CU Boulder campus. Sites were labeled *A*–*S* going from the most southerly to most northerly sampled location, except for the isolation (*Isolation*) and administrative buildings (*Admin*). If specific locations received flow from multiple targeted structures, then the primary location was listed first with other contributing flows presented in brackets [e.g., *A*(*B*), *E2*(*CBA*), and *G*(*FEDCBA*)]. The autosamplers withdrew approximately 10-l over 24-hour, and triplicate 50-ml subsamples were collected daily from each autosampler between 7:00 a.m. and 12:00 p.m. After transport to the laboratory, these samples were spiked with bovine coronavirus as an internal process control, and the sample was concentrated using electronegative filter pipettes (Innovaprep, Drexel, MO) and the RNA was extracted with a commercially available kit (RNALink, Thermo Fisher, Waltham, MA), respectively. After quantifying the extracted RNA on a Qubit (Thermo Fisher), the “SENB+” pipeline was run. “SENB+” consists of a reverse-transcription quantitative polymerase chain reaction (RT-qPCR) multiplex TaqMan assay used to detect SARS-CoV-2 N (N2), SARS-CoV-2 E, bovine coronavirus, and genogroup II F+ RNA bacteriophage. All RT-qPCR analyses were performed on a QuantStudio 3 (Thermo Fisher). A daily process blank (bovine coronavirus spiked ultrapure water) was run with the wastewater samples, and at least three no-template controls were used on the RT-qPCR plate.

For the Spring campaign, sample collection and processing was conducted as described previously from 7 February 2021 until 1 May 2021 (Tables S1–S8, Supporting Information; Reeves et al. 2021). Sampling was suspended February 11th–18th because of an extended period of cold temperatures freezing the inlets to sample stations and causing hazardous exposure conditions to the sample collectors. During this Spring campaign, a single modification to the continuous samplers was included by adding a piece of 0.5-inch inner diameter PVC tubing with threaded fixtures attached and cemented to the small outlet of the jerrycan reservoir; this tube inlet was raised above the full volume line to eliminate episodic leakages. SARS-CoV-2 RNA was concentrated, extracted, and quantified using the mentioned SENB+ pipeline, with the modification that genogroup II F+ RNA bacteriophage was replaced with PMMoV as a human fecal indicator (Tables S9 and S10, Supporting Information; Symonds et al. 2019). Recovery of the bovine coronavirus spike-in was consistent throughout (Figures S1 and S2, Supporting Information), and the resulting correlation between the envelope and nucleocapsid targets remained high for the Spring campaign (Pearson r = 0.93; Figure S3, Supporting Information). All processing data is included in the supplementals. Within this study, data from the Fall campaign were taken directly from supplementals of Reeves et al. (2021; Table S12, Supporting Information).

### Data processing pipeline

Raw RT-qPCR data was processed using QuantStudio 3 and 5 Design and Analysis Software (Thermo Fisher, v. 1.5.1) on a Windows 10 machine (Microsoft, Redman, WA). Default parameters were used for cycle threshold (Ct) determination, except for when automatic thresholds needed to be manually corrected in accordance with software guidelines. Data from wells that had no content or in which bovine coronavirus did not amplify (all fluorescence measurements of zero) were excluded from analysis. Further automated postprocessing in R (v. 4.1.2; R Core 2022; Supplemental File 1) included the following: calculating allowable differences in Ct value between replicate amplifications according to the Poisson distribution, excluding amplifications that violated those thresholds (de Ronde et al. 2017), and omitting any standard when at least one of the replicates displayed a greater Ct value than the associated negative controls. After establishing a linear standard curve based first on the “A,” “B,” and “C” standard levels, higher and lower dilutions were omitted when their mean Ct values fell outside the 95% confidence limits of the linear curve (Figure S4, Supporting Information). The lowest-concentration standard level remaining after cleaning for each plate and target established the limit of quantification (LoQ). The limit of detection (LoD) was set at the maximum cycle (the 40th cycle) or lower if nonspecific amplification was observed in the negative control reactions. The final processed standard curves were generated for each plate and every target and used in quantification. Target copies per liter wastewater were calculated from target copies per RT-qPCR reaction using the following equation:

(1) \begin{eqnarray*}
Target\ copies/L\ = \frac{{gene\ copies}}{{2.5\ \mu L\ extracted\ RNA}}\ *\frac{{50\ \mu L\ extracted\ RNA}}{{230\ \mu L\ concentrate}}\\
\quad*\frac{{X\ \mu L\ concentrate}}{{Y\ L\ wastewater\ sample\ processed}}.
\end{eqnarray*}

### Weekly norovirus and influenza testing

Norovirus and influenza RNA were detected from the same sample RNA extract used to test for SARS-CoV-2. Testing for those viruses began on 9 April 2021, approximately 2 months after the start of the Spring campaign. For the remaining month of the campaign, testing occurred weekly. Samples that were collected on Monday (or Tuesday, when Monday samples were not collected) were primarily analyzed by Friday of the same week. Monday (or Tuesday) samples taken in the Spring campaign prior to the launch of the norovirus and influenza assays were tested retroactively in May 2021.

Norovirus and influenza were tested for with separate one-step RT-qPCR assays. The norovirus assay targeted norovirus genogroups I, II, and IV (GI, GII, and GIV). The influenza assay targeted influenza types A and B. Both assays targeted the bovine coronavirus spiked internal process control and employed norovirus and influenza specific primers and probes (Table S10, Supporting Information). For both assays, RT-qPCR amplifications were performed in 20-µl reactions including 5 µl of TaqPath^TM^ One-Step Multiplex Master Mix (Thermo Fisher) and 2.5 µl of RNA template. The norovirus reaction additionally comprised 0.025 µl of each 400-uM GI and GIV primers, 0.03 µl of 400-µM GII primers, 0.05 µl of 200-µM bovine coronavirus primer, 0.04 µl of each 100-uM probe, and 12.21-µl nuclease-free water. The influenza reaction included 0.025 µl of each 400-µM A and B primer, 0.05 µl of 200-µM bovine coronavirus primer, 0.04 µl of each 100-µM probe, and 12.21-µl nuclease-free water. Both norovirus and influenza plate runs were performed on a QuantStudio 3 (Thermo Fisher) according to the following program: UNG incubation at 25°C for 2 minutes, reverse transcription at 53°C for 10 minutes, polymerase activation at 95°C for 2 minutes, and amplification in 40 cycles of denaturing at 95°C for 3 seconds, annealing at 55°C for 15 seconds, elongating at 60°C for 15 seconds, and subsequent detection of the FAM, VIC, ABY, and JUN fluorophores.

Reactions amplifying sample RNA were performed in technical triplicates. Each run included at least one no-template control reaction. Serial dilutions of single-stranded RNA (bovine coronavirus) and DNA (norovirus and influenza) were used for standard curve quantification. A fresh standard dilution series was created before each run. Raw RT-qPCR data was processed and cleaned as described for the SARS-CoV-2 data. Of note, the influenza and norovirus standard curves performed poorly (Figure S5, Supporting Information). Their performance does not diminish our results as absolute quantification was not critical to interpretation of the norovirus and influenza data. However, future studies should optimize these assays.

### Exploring norovirus GII spikes

Over the course of testing weekly samples, two relatively high norovirus GII signals were noted for the *Admin* (23 February 2021) and *P* (8 March 2021) structures. To better investigate these spikes, representative samples from surrounding days were tested using the described norovirus assay, with the notable exception that larger differences in Ct value between replicate amplifications were allowed. Greater tolerance was required to not artificially exclude data resulting from the smaller size of this dataset.

### Decreased sampling frequency exploration

To compare the accuracy of daily to less frequent sampling scenarios in both times of high community spread (Fall) and times of low/sporadic community spread (Spring), the Fall and Spring data were subsampled by weekday according to the following sampling schemes: Monday, Wednesday, Friday (MWF); Monday only (M), and Saturday only (S). The reduced sampling scheme excluded actual measured values (e.g., MWF excluded Tuesday, Thursday, Saturday, and Sunday samples) and replaced those excluded values with the most previous measurement (e.g., Tuesday values were replaced by Monday values). For each of these schemes, differences between the replaced value and the value that it replaced were calculated daily for each sampling site. For each residential structure, the differences from each scheme were summed for the entire semester and normalized over the semester-total copies per liter wastewater for that structure. A pairwise Wilcoxon test was then implemented in R using the rstatix (version 0.7.0) package to compare the ratios for all residential structures between the Fall and Spring semester, grouped by the subsampling schedule (Kassambara 2020).

Additionally, four partitions of viral prevalence that coincide with those used during monitoring to inform pandemic response efforts were defined. Partition I (P1) correspond with SARS-CoV-2 copies per liter wastewater values less than 1,000, P2 with values between 1,000 and 10,000, P3 with values between 10,000 and 100,000, and P4 with values above 100,000. To determine how well each reduced sampling scheme correctly identified the partition for every residential structure in the Fall and Spring semesters, the SARS-CoV-2 copies per liter wastewater data were replaced with the corresponding discrete variables of P1, P2, P3, and P4. Raw counts of days for which the partition was misidentified were compared between Fall and Spring using a Fisher’s exact test.

### Pipeline dilution analysis

The placement of a group of residential structures along the same sewer network enabled analyzing SARS-CoV-2 loads through a sewer network (Fig. 3A). The flow-path connected structures include *A*, *B(A)*, *C*, *D*, *E(CBA)*, and *F*. Structure *A* contributes upstream of *B*, and structures *A*, *B*, and *C* contribute upstream of *E*. Resulting from the placement and availability of manholes, and as indicated by their parentheticals, wastewater output from *B* combined with *A* prior to sampling at *B(A)*. Wastewater output from E was collected after combination with wastewater from *A*, *B*, and *C* at *E(CBA)*. Wastewater was additionally collected at site *G*, a point at which flows from all six of the identified structures combined. Translating SARS-CoV-2 concentration values into mass loads at these sites in the absence of wastewater flow values required multiplying envelope copies per liter wastewater by the number of residents contributing to the flow, using population as a flow proxy. Thus, each viral load value was represented as a population-normalized copy number.

The daily viral loads contributed by only B, E, and F and D (to be referred to together as FD) was calculated by subtracting known upstream signals from the known, combined, downstream signals as follows:

(2) \begin{eqnarray*}
{\mathrm{Po}}{{\mathrm{p}}}_{\mathrm{B}}*{\left[ {{\mathrm{SARS}} - {\mathrm{CoV}} - 2} \right]}_{\mathrm{B}} &=& {\mathrm{Po}}{{\mathrm{p}}}_{{\mathrm{B}}({\mathrm{A}})}*{\left[ {{\mathrm{SARS}} - {\mathrm{CoV}} - 2} \right]}_{{\mathrm{B}}({\mathrm{A}})}\\
&&\quad - {\mathrm{Po}}{{\mathrm{p}}}_{\mathrm{A}}*{\left[ {{\mathrm{SARS}} - {\mathrm{CoV}} - 2} \right]}_{\mathrm{A}}.
\end{eqnarray*}

(3) \begin{eqnarray*}
{\mathrm{Po}}{{\mathrm{p}}}_{\mathrm{E}}*{\left[ {{\mathrm{SARS}} - {\mathrm{CoV}} - 2} \right]}_{\mathrm{E}} &=& {\mathrm{Po}}{{\mathrm{p}}}_{{\mathrm{E}}({\mathrm{CBA}})}*{\left[ {{\mathrm{SARS}} - {\mathrm{CoV}} - 2} \right]}_{{\mathrm{E}}({\mathrm{CBA}})}\nonumber\\
&&\quad - {\mathrm{Po}}{{\mathrm{p}}}_{{\mathrm{B}}({\mathrm{A}})}*{\left[ {{\mathrm{SARS}} - {\mathrm{CoV}} - 2} \right]}_{{\mathrm{B}}({\mathrm{A}})}\nonumber\\
&&\quad -{\mathrm{Po}}{{\mathrm{p}}}_{\mathrm{C}}*{\left[ {{\mathrm{SARS}} - {\mathrm{CoV}} - 2} \right]}_{\mathrm{C}}.
\end{eqnarray*}

(4) \begin{eqnarray*}
&& {\mathrm{Po}}{{\mathrm{p}}}_{{\mathrm{FD}}}*{\left[ {{\mathrm{SARS}} - {\mathrm{CoV}} - 2} \right]}_{{\mathrm{FD}}}\nonumber\\
&&\qquad = {\mathrm{Po}}{{\mathrm{p}}}_{{\mathrm{G}}({\mathrm{FEDCBA}})} *{\left[ {{\mathrm{SARS}} - {\mathrm{CoV}} - 2} \right]}_{{\mathrm{G}}({\mathrm{FEDCBA}})} - {\mathrm{Po}}{{\mathrm{p}}}_{{\mathrm{E}}({\mathrm{BCA}})}\nonumber\\
&&\qquad *{\left[ {{\mathrm{SARS}} - {\mathrm{CoV}} - 2} \right]}_{{\mathrm{E}}({\mathrm{BCA}})} - {\mathrm{Po}}{{\mathrm{p}}}_{{\mathrm{E}}2}*{\left[ {{\mathrm{SARS}} - {\mathrm{CoV}} - 2} \right]}_{{\mathrm{E}}2}.
\end{eqnarray*}

These estimations were then compared to the Spring medical services data for B, E, and FD to explore accuracy.

### Correlation between residential structures

To investigate whether correlations in SARS-CoV-2 viral shedding between residential structures would be more apparent in times of low than high community spread, the R package corrplot (v. 0.92) was used to calculate the significance of correlations between all sampling sites for both the Fall and Spring semester (Wei et al. 2017). A Spearman’s correlation was used to avoid Pearson correlation assumptions of normality and lack of outliers. Missing data was deleted from correlations on a pairwise basis, although data may not be missing completely at random resulting from known sampling patterns (Little’s test, Fall semester: *P* = .0873; Spring semester: *P* = .245). Significance tests and plotting were also implemented in corrplot, with significance levels = 0.001, 0.01, and 0.05. A dendrogram was constructed in R using hierarchical clustering with Ward’s method and compared to the physical proximity of residential structures around CU Boulder.

To investigate whether a time delay would reveal correlations in the case of one residential structure driving transmission to another, a cross-correlation was used to calculate the log_10_ adjusted correlation for every residential structure compared to that of every other residential structure with a time lag from 0 to 14 days. The mean for each time lag was calculated, and this procedure was repeated for an autocorrelation. Comparisons were made between the autocorrelation and cross-correlation values, as well as between the Fall and Spring semester.

## Results and discussion

### Summary of spring dataset

**Figure 1.**
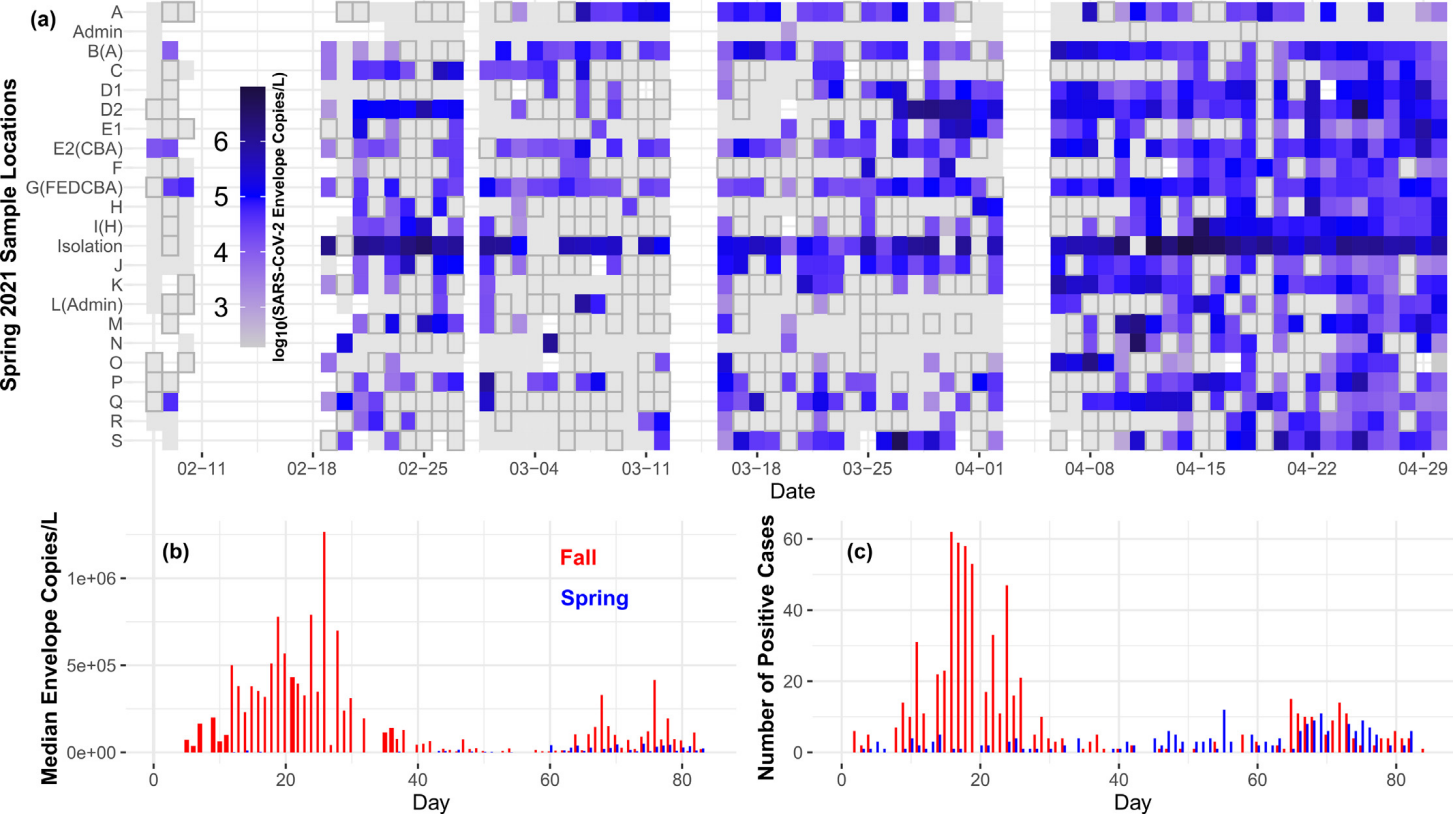
(a) Heatmap of log_10_ SARS-CoV-2 envelope copies per liter wastewater concentrations across the Spring campaign. Intensity of the concentration is depicted from light to dark blue, with white representing samples not taken (area between 02–11 and 02–18 represents the significant cold temperature event that caused the suspension of sample collection). Outlined gray squares indicate samples with SARS-CoV-2 envelope copies detected below the LOQ, whereas gray squares without an outline indicate no SARS-CoV-2 envelope copies detected. The sampling campaign concluded on 1 May 2021. (b) Comparison of the Fall (red) and Spring (blue) median envelope concentrations across all sites (excluding the structures that housed isolating infected students). (c) Comparison of the Fall (red) and Spring (blue) daily infections detected through medical services (Table S12, Supporting Information). For both (b) and (c), the thicker bars for the Fall campaign indicate samples without a direct comparison in the Spring resulting from the suspension of sampling. The alphabetical designation is arranged spatially, with A being the furthest South and S being the furthest North sample station on the sewage network.

The Spring 2021 CU Boulder surveillance effort presented in this study monitored a period of low on-campus prevalence of SARS-CoV-2 and continued from the successful implementation of the Fall 2020 campaign when SARS-CoV-2 prevalence was higher (Reeves et al. 2021). During this period of reduced caseloads, the Spring campaign was able to identify individual infections within the on-campus residential structures (Fig. 1a). Additionally, median daily concentrations of SARS-CoV-2 in wastewater were substantially lower in the Spring than in the Fall, with the overall mean number of SARS-CoV-2 envelope copies per liter wastewater from the entire Spring dataset nearly five times lower than that from the Fall (Fig. 1b). This consistently lower concentration agreed with the lower population prevalence detected through the on-campus medical services diagnostic data (Fig. 1c). However, toward the end of the Spring campaign, more infections were being detected and recorded, with only a modest increase in the median SARS-CoV-2 copies per liter wastewater concentration when compared to the Fall (Fig. 1b). Speculatively, detection, contact tracing, and isolation (i.e., removal from detection through wastewater) became more routine in the Spring, vaccines became more available toward the conclusion of the sampling campaign, and human behavior and on-campus occupancy shifted, all likely contributing to this disconnect (Bivins and Bibby 2021). Overall, these Fall and Spring semesters of the campaign monitored during two uniquely informative aspects of pandemic behavior—community spread and sporadic low-level infections. Comparing these two semesters allows exploring both the utility of the data and the required resource commitment during key phases of a public health emergency.

### Temporal resource commitment—effect of decreased sampling frequency

**Figure 2.**
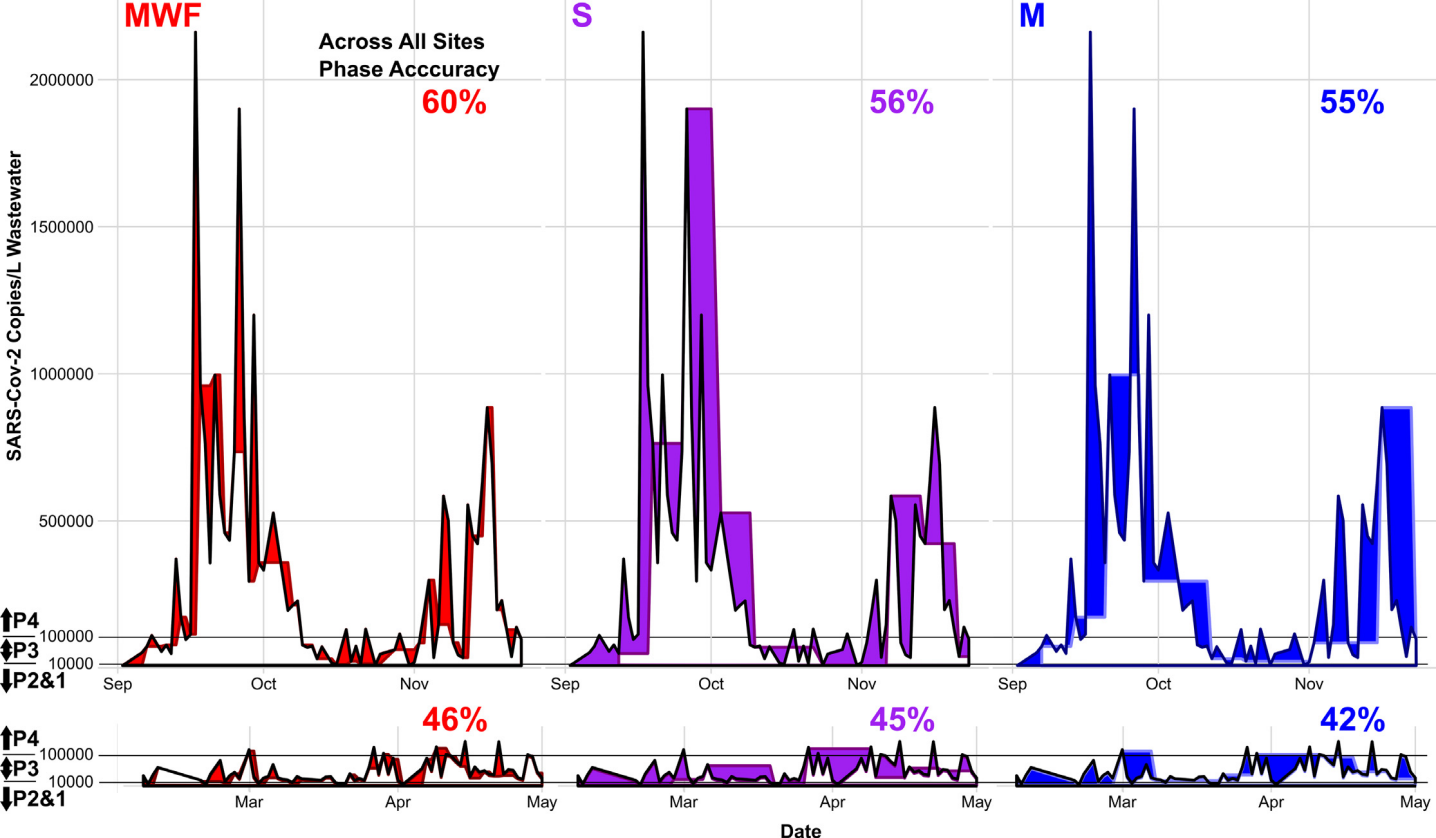
Site G(FEDCBA) time series of SARS-CoV-2 envelope copies per liter wastewater for the as-measured values (black line) and reduced sampling scenarios [Monday only (M, blue); Monday, Wednesday, Friday (MWF, red); and Saturday only (S, purple)] for both the Fall and Spring semesters. The areas shaded with color highlight the difference between the as-measured value and the predicted value from the subsampling. The four partitions of viral prevalence were defined to correspond with SARS-CoV-2 envelope copies per liter wastewater values of less than 1,000 (Partition 1, P1), between 1,000 and 10,000 (P2), between 10,000 and 100,000 (P3), and above 100,000 (P4). The concentration levels for the partitions are presented on the *y*-axis. All sites are presented on a log-scale in Figure S6 (Supporting Information). The partition accuracy represents the percentage of all pairwise observations between the as-measured values and reduced sampling scenarios that match partitions for the respective semesters.

Across all building-level campus WBS campaigns, sampling frequencies have ranged from weekly to daily (Betancourt et al. 2021, Gibas et al. 2021, Karthikeyan et al. 2021, Scott et al. 2021). Less frequent sampling schedules may be necessitated by the financial and labor cost associated with daily sample collection, processing, and analysis. Whether reduced sampling schedules are informative during times of sporadic infection/low community spread (e.g., Spring) or times of high community spread (e.g., Fall) remains less explored (Fig. 2). Therefore, subsampling the daily nature of the reported campaign into artificial MWF, M, and S campaigns enables exploring the effort-to-accuracy required. For all subsampling scenarios, the mean difference between the concentration value that was excluded, and the value used to replace was higher in the Fall semester than in the Spring (Wilcoxon rank order test supported unequal means). When these differences were normalized over the concentration measurements that were excluded, the two semesters were indistinguishable (Wilcoxon rank order test support of equal means). Taken together, the higher caseload and resulting concentrations of SARS-CoV-2 within wastewater during the Fall semester resulted in a higher absolute difference between the replaced and the actual recorded daily value, but not necessarily the normalized, when compared to the Spring. Detecting consistency in the normalized difference builds confidence that the methods and approach did not bias or unevenly influence the monitored results, notably when experiencing substantially different phases in community spread.

To test the monitoring accuracy of reduced sampling schedules further, virus prevalence was first assigned discrete partitions [SARS-CoV-2 envelope copies per liter wastewater values of less than 1,000 (Partition 1, P1), between 1,000 and 10,000 (P2), between 10,000 and 100,000 (P3), and above 100,000 (P4)]. Although this analysis is subject to variability depending on the numeric value, these thresholds were defined according to their perceived relevance to pandemic response efforts. This process enabled calculating the number of replaced-value days (i.e. days artificially substituted by the previous measurement) in each scenario that correctly partitioned with the excluded sample (Fig.   2B). Across all three sampling schedules, the Spring semester displayed a lower partitioning accuracy than the Fall (46%, 45%, and 42% accurate in the Spring; 60%, 56%, and 55% in the Fall for the MWF, S, and M subsampling, respectively). The frequency of inaccurate partition assignments was significantly different between semesters (Fisher’s exact test, *P*-value = 2.2 × 10^−16^). Although the Fall semester displayed higher overall mean differences in values, the higher accuracy in partitioning during the Fall likely originates from two features of infection dynamics. First, whereas outbreaks in the Spring semester were highly sporadic—and often contained quickly—community transmission during the Fall semester tended to be more sustained, increasing the likelihood that the subsequent day’s sample would be at or near the concentration value of the previous day. Second, the viral concentration in wastewater in the Fall was often measured to be well above the threshold defined for the highest level of concern, minimizing the effect of variation when the data are transformed into discrete partitions. These data support the counterintuitive hypothesis that increased temporal sampling resolution may have decreased utility during times of high community transmission compared to times of low-to-sporadic transmission. Notably, 3-day sampling schedule (accuracy of 60% for Fall and 45% for Spring) displayed a similar accuracy to the single-day sampling (accuracy of 55% and 54% for Fall and 45 and 42% for Spring). Therefore, if resources are constrained, then single-day sampling should be sufficient when considering accuracy alone.

### Spatial resource commitment—pipeline dilution analysis

**Figure 3.**
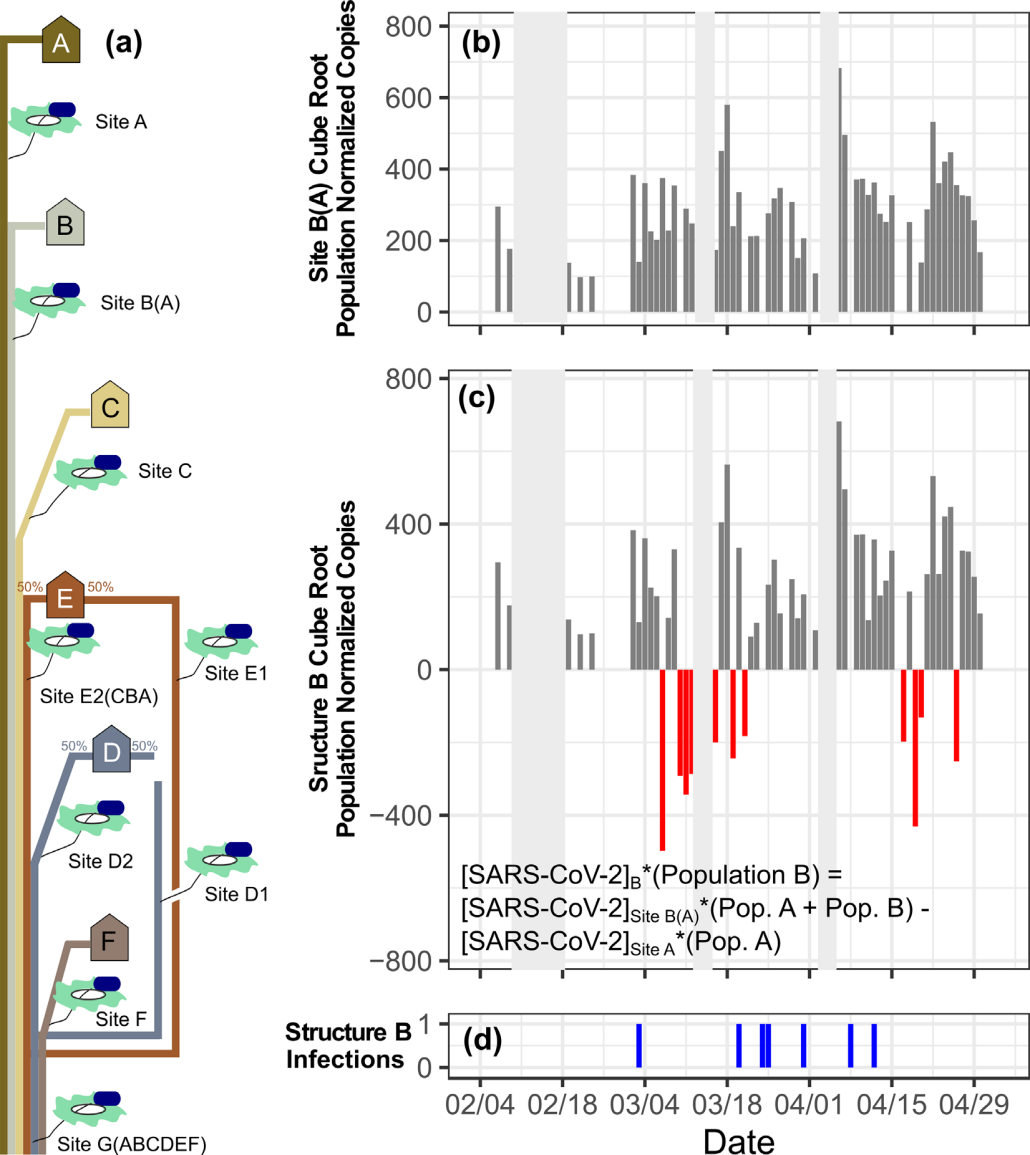
**(a)** Cartoon depicting the monitored residential structures (⟰), the sewer sampling locations (⦸), and combined flow paths of the considered sewer network. **(b)** Daily wastewater viral loads (population-normalized envelope copies, cube rooted) detected at site *B(A)*. **(c)** Loads contributed by structure *B* estimated by assuming upstream viral signals are reliably transported downstream (with an example equation to arrive at the amount of SARS-CoV-2 RNA uniquely contributed by structure *B*). Gray regions indicate times in which the sampling was suspended. Red bars highlight those predictions that are negative. **(d)** The associate infections detected within *B* structures by medical services. All considered structures are presented in Figure S7 (Supporting Information).

In addition to temporal sampling effects influencing the accuracy of the predicted monitored value, spatial effects may result from the structure of the sewer network. Specifically, infections detectable at upstream locations may not be detectable as wastewater travels downstream. Notably, exploring whether SARS-CoV-2 concentrations were recoverable downstream in the monitored sewer network using conservation-of-mass naïve estimates resulted in negative concentration predictions for monitored structures *B*, *E*, and *FD* (Fig. 3). Negative values represent upstream population-normalized copy numbers exceeding downstream numbers. This result suggests that either estimations of flow were inaccurate or upstream viral signals were not reliably transported and detected downstream. SARS-CoV-2 RNA has been observed to follow a first-order decay rate in wastewater, and those viral signals may have been lost while traveling because of in-pipe processes such as adsorption and biodegradation, an area of research need (Ahmed et al. 2020, Weidhaas et al. 2021, Kostoglou et al. 2022, Shi et al. 2022). Additionally, the sample material may have only been erratically recovered by the autosamplers resulting from poor mixing within the sewer channel and/or occasional autosampler failures (e.g., solid masses such as toilet paper knocking the inlet strainer out-of-stream or floating fecal matter above the water level) amongst other known processing constraints (Saguti et al. 2021). Overall, the *B*, *E*, and *FD* population-normalized copy profiles approximate their parent combined-structure (*B(A)*, *E(CBA)*, and *G(FEDCBA)*) profiles. This similarity indicates that the daily upstream values subtracted out were relatively low and sporadic, consistent with the epidemiological character of the SARS-CoV-2 pandemic on campus during the Spring semester. Larger caseloads like those observed in the Fall semester may have resulted in stronger and clearer conveyances of upstream signals downstream, but could not be explored further as the sampling sites of interest were primarily launched post 31 October 2020. Under the conditions of our analysis, distinguishing contributions from infections in upstream structures further downstream in the pipe network during a phase of low and sporadic infection was unreliable, as demonstrated by the resulting negative population-normalized values.

When relating the population-normalized SARS-CoV-2 signal to detected infections upstream rather than the reported wastewater concentrations, the signal appeared to reflect infections within the monitored structure. Throughout the Spring, positive population-normalized values occurred for 26 out of 42 observations when paired with an infection detected by medical services. However, when compared to the 126 of 180 observations displaying positive population-normalized values, the success ratio when paired with infections does not display a significant overrepresentation (*P* = .06 for a hypergeometric distribution test). Overall, a number of limitations biased this analysis, including the need to use population as a flow proxy, the inability to accurately account for flows such as nonresidential dining-services related flows and a graywater recirculation system, and uncertainty about in-pipe hydraulic and biological processes, such as the accumulation of SARS-CoV-2 RNA in sewer biofilms, and their effect on the measured downstream viral concentration (Morales Medina et al. 2022). Utilizing a within-wastewater normalizing factor, such as the monitored PMMoV signal, may help to account for these factors, but was considered outside the scope of the current analysis because it was not used within the reporting of the initial results. Additionally, these errors may be more pronounced at upstream locations with lower flow. Overall, understanding how the sewer conveyance network itself modifies upstream detected signals as it flows downstream is important when discriminating between specific structures or watersheds, as demonstrated by the potential spatial bias detected in the Spring semester and the expanding active research in this area (Kuhn et al. 2022, Vallejo et al. 2022, Zulli et al. 2022).

### Utility of data—PMMoV standardization

**Figure 4.**
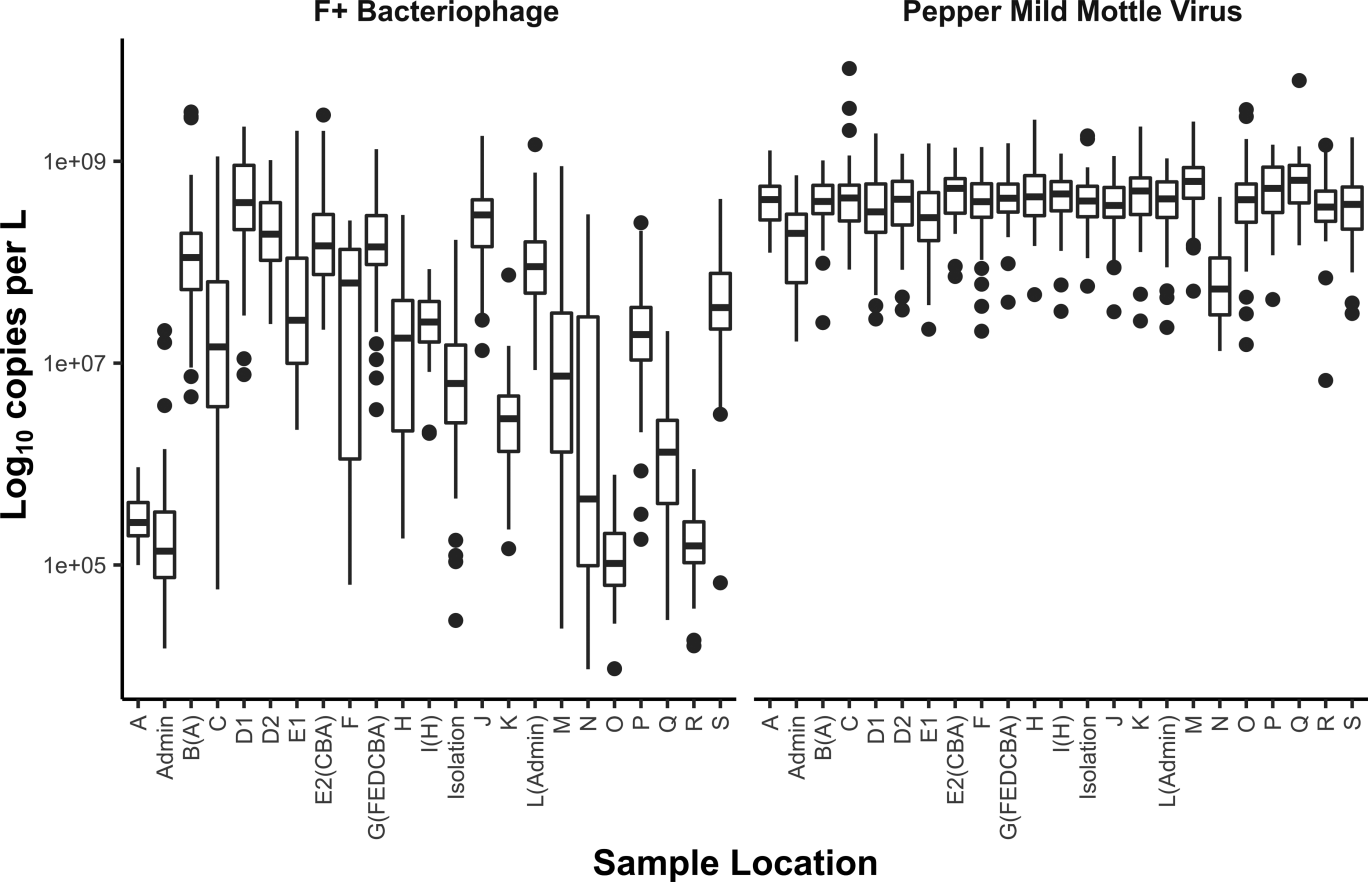
Quantile plots of the genomic copies per liter wastewater values from Fall 2020 monitoring of F+ bacteriophage and Spring 2021 monitoring of PMMoV.

A potential approach to standardize the influence of both the sewer network and technical processing is establishing a reliable within-sample sewage indicator. During the Fall semester, F+ bacteriophage was used as a human fecal indicator, but it displayed variability over several orders of magnitude between different sample locations and days (Reeves et al. 2021). Therefore, for the Spring semester, F+ bacteriophage was replaced by PMMoV (Rosario et al. 2009, D’Aoust et al. 2021). Wastewater concentrations of PMMoV in this study were generally above the LoQ and displayed higher consistency than was observed with F+ bacteriophage (Fig. 4). Out of 46 occasions (3% of the total 1532 measurements) in which PMMoV was found to be below the LoQ in wastewater (not including the 183 nondetects), 17 of these were from the *Admin* site or residential structure *N*. These LoQ violations likely resulted from the low and fast flow conditions at *Admin* and *N*, respectively (George et al. 2022). Therefore, PMMoV generally functioned more consistently as a human fecal indicator than F+ Bacteriophage (noting, though, that they were not tested on the identical dataset). Supporting previous findings and applications, these PMMoV values should be considered in the future to potentially normalize the SARS-CoV-2 data (D’Aoust et al. 2021, Wolfe et al. 2021, Holm et al. 2022).

### Spatial resource commitment—correlation between residential structures

A correlation between residential structures was conducted to determine whether the sporadic transmission or the community spread experienced in the Spring or Fall semester, respectively, would allow for the detection of one residential structure driving transmission to another using solely wastewater data. During the low/sporadic community transmission in the Spring, the correlations are nearer to zero than those observed for the Fall (Figure S8, Supporting Information). By clustering near zero, these values trend toward statistical independence, a trait likely representative of sporadic infection incidences. During the Fall semester, the community spread caused more nonspecific clustering, with the majority of residential structures displaying positive correlations. However, direct correlations may not be well suited to capture one residential structure driving transmission to another because of the following: (1) transmission from one population to another is a discrete event with infections potentially passed between structures, (2) high correlations would be more reflective of concurrent transmission, indicating that individuals in each residential structure are infected from the same source, and (3) the nature of the transmission event, such as community spread or sporadic infections, may mask the utility of correlation. Therefore, cross-correlations between each residential structure were calculated to determine whether the event of one driving transmission to another could be better captured through the introduction of a 0–14-day time-lag (Figure S9, Supporting Information). The higher correlation between residential structures in the Fall is also detected in these cross-correlations. However, this analysis is limited by the fact that a single lag time could not be calculated to capture transmission from one residential structure to another, as this relationship is determined by resident social behavior. Combined, these correlation analyses highlight that WBS alone does not fully recover noticeable trends in residential behavior at such a fine resolution, requiring other public health initiatives such as robust contact tracing methods to better understand the dynamics of SARS-CoV-2 concentrations in wastewater.

### Utility of data—weekly norovirus and influenza testing

**Figure 5.**
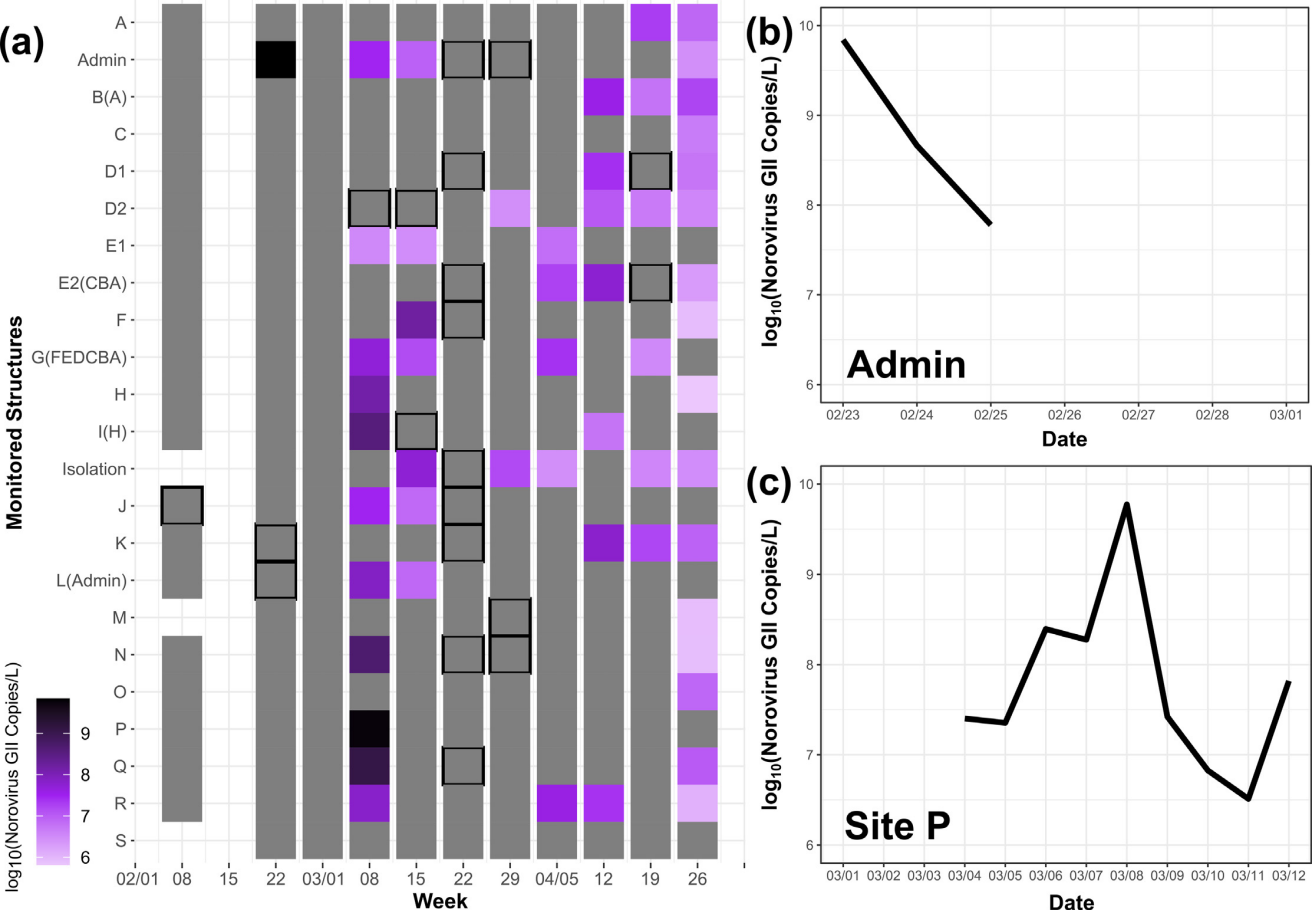
(a) Log10 norovirus GII copies per liter wastewater values measured weekly over the Spring 2021 semester. Gray indicates sampled but below LOD; boxed gray indicates above LOD but below LOQ. Log_10_ norovirus GII copies per liter wastewater values of (b) the *Admin* structure in days within the week of 2/22 and (c) the P structure in days surrounding 3/08. Absent values for (b) 2/26–3/01 (with the exception of 2/28—no results from any samples) and (c) 3/01–3/03 are below the LOD; error bars are not presented because only technical replicates were run.

The utility of WBS explored above for SARS-CoV-2 is generalizable for other pathogens of interest. For example, the norovirus and influenza signals found in campus wastewater largely reflected the trends reported in Colorado Department of Public Health and Environment (CDPHE) and Centers for Disease Control and Prevention (CDC) disease surveillance efforts, with both being primarily absent. Notably, distinct norovirus peaks were observed throughout the monitoring period (Fig. 5).

The detection of the two norovirus spikes on February 23rd for structure *Admin* and March 8th for structure *P* triggered exploration into how frequently testing needs to occur to reliably catch those spikes. The *Admin* samples collected on the days surrounding February 23^rd^ and the *P* samples collected on the days surrounding March 8th were tested for norovirus and used to perform a subsample analysis as previously described for SARS-CoV-2 data above, but comparing samples withdrawn (1) every day, (2) only Mondays, Wednesdays, and Fridays (MWF), and (3) only Tuesdays and Thursdays (TTh). Analysis surrounding the February spike was discarded resulting from a lack of *Admin* samples collected prior to February 23rd. For structure *P*, whereas the spike in concentration is found on the daily and MWF schedules, the increase is not noted on the TTh schedule simply because a sample from that specific Wednesday is missing. This result supports daily testing when time and resources allow to ensure infrequent norovirus spikes during low overall prevalence with the potential to lead to community spread are found, corroborating the previous analysis for the SARS-CoV-2 data.

With recent developments based on SARS-CoV-2 detection and considered care in application, WBS may inform on key outbreaks of other respiratory viruses including influenza, overcoming previous limitations (Prevost et al. 2015, Wolfe et al. 2022). The CDPHE reported historically low levels of influenza activity during the 2020–2021 flu season in Colorado (27 September 2020–22 May 2021) resulting from COVID-19 mitigation measures (CDPHE 2021). A total of 34 hospitalizations were reported, approximately 1% of the 3546 hospitalizations reported during the 2019–2020 season and 10% of the 363 hospitalizations reported during the 2006–2007 season (the lowest number of hospitalizations recorded prior to 2020; CDPHE 2021). Outpatient and emergency department providers also did not experience any influenza-like illness (ILI) activity peaks during the 2020–2021 season (maximum percentage of ILI-coded visits with outpatient providers was 1.67%), compared to three peaks in the 2019–2020 season (maximum percentage of ILI-coded visits with outpatient providers was 8.3%; CDPHE 2021). Consistent with this reporting, minimal influenza RNA was found in campus wastewater via testing of weekly samples. The few instances of influenza detection were not temporally sustained and may be explained by PCR plate artifacts, indicating a lack of community spread. This reflection of the medical findings supports the ability to understand community influenza burden through wastewater testing but requires further validation during a year in which influenza is at a higher prevalence.

## Conclusions

Linking the resource requirements for effective WBS to the prevalence of the virus within a community informs operators on required sampling frequency and the effort overall required to maintain effective campaigns. For example, monitoring during low-viral prevalence (i.e., Spring 2021) introduced unique resource constraints. Although overall caseloads were lower in the Spring semester, WBS was still successfully implemented to capture individual cases of COVID-19 and monitor overall community transmission of SARS-CoV-2. Additionally, the effect of simulated decreased temporal sampling resolution during times of the lower overall transmission in Spring 2021 was found to display lower error in estimating the daily copies per liter wastewater when compared to times of high transmission in Fall 2020. However, Spring additionally displayed a lower overall accuracy at tracking the relevant stage of community transmission within the individual structures. Therefore, an inverse resource commitment is required, with a community experiencing a lower SARS-CoV-2 prevalence requiring a more frequent sampling schedule to ensure the dynamics of the virus are accurately represented during the crucial introduction and initial spread phases. When considering the spatial deployment of resources, error was also noted in attempting to infer the viral load contribution from a single residential structure in a wastewater stream with mixed inputs in the spatial analysis, with this approach being deemed ineffective because several days were calculated to have a negative viral load in downstream structures. Therefore, samples should be withdrawn as near to the monitored subsection of the community as possible, because combined flows may introduce errors in interpretation. However, even with a near building-level resolution across a university campus, correlations between residential structures were nearer to zero overall for the Spring semester when compared to the Fall semester. This indicated that although correlations were relatively strong when high-prevalence of SARS-CoV-2 was detectable across campus, the social factors behind the spread of the virus during low prevalence could not be explained by relationships in wastewater concentrations alone. Several of these WBS challenges associated with low-prevalence were also noted for norovirus and influenza, although the data for these viruses largely reflected the low-community spread noted overall by the CDPHE data. As WBS application expand to effectively monitor new targets, continuing to understand and optimize the spatial and temporal resource commitment required to achieve data supportive of specific interventions of viral spread within a population ensures the maturation of WBS into a cost-effective, widely accessible, and adaptable technique.

## Supplementary Material

xtac024_Supplemental_Files

## Notes

*Conflict of interest statement*. The authors declare no competing interests in the publication of this article. Cresten Mansfeldt began consulting for Flambeau RapidX in May 2022, well after the completion of the campaign. Additionally, the initial campaign was subjected to an internal review and was determined to not fall under the purview of regulations that cover human-subject studies of a typical IRB. Anonymity was preserved by masking the individual sites with identifiers rather than structure names.
