# Supplementary material for: Effectiveness of building-level sewage surveillance during both community-spread and sporadic-infection phases of SARS-CoV-2 in a university campus population
Source: FEMS Microbes. 2022 Sep 24;3:xtac024. doi: 10.1093/femsmc/xtac024 (PMC10117889; doi:10.1093/femsmc/xtac024)
Supplement: xtac024_Supplemental_Files [file xtac024_supplemental_files.zip › 20220807_Johnson_et_al_Supplemental Figures_Final_Corrected.docx]

**Supplemental Figures**

**Effectiveness of building-level sewage surveillance during both community-spread and sporadic-infection phases of SARS-CoV-2 in a university campus population**

**Authors:**

William Johnson^1,2*^; Katelyn Reeves^1,2*^; Jennifer Liebig^3^; Antonio Feula^3^; Claire Butler^2^; Michaela Alkire^2^; Samiha Singh^2^; Shelby Litton^2^; Kerry O'Conor^2^; Keaton Jones^2^; Trace Shimek^2^; Julia Witteman^2^; Sampling Team Collaborators; Kristen K. Bjorkman^3^; Cresten Mansfeldt^1,2†^

1. University of Colorado Boulder, Department of Civil, Environmental, and Architectural Engineering, 1111 Engineering Drive, Boulder, CO 80309, United States

2. University of Colorado Boulder, Environmental Engineering Program, 4001 Discovery Dr, Boulder, CO 80303, United States

3. University of Colorado Boulder, BioFrontiers Institute, 3415 Colorado Avenue, Boulder, CO 80303, United States

Supplemental Figure 1. Concentration (log scale) of the bovine coronavirus spike-in across all sites for all samples

Supplemental Figure 2. Concentration (log scale) for the process blank with a bovine coronavirus recovery spike-in

Supplemental Figure 3. SENB+ Cq values for the A-F standard curves. Standard concentrations described in Supplemental Table 10

Supplemental Figure 4. Scatterplot comparing Nucleocapsid (N) and Envelope (E) copies per L wastewater across all spring samples after processing pipeline

Supplemental Figure 5. Cq values for the standard curves for the norovirus and influenza assays. Standard concentrations described in Supplemental Table 10

Supplemental Figure 6. Copies (Envelope) per L wastewater for full data and simulated reduced sampling schedules for all sites between fall and spring, dashed lines indicate thresholds of 1000, 10,000, and 100,000.

Supplemental Figure 7. Plots depict daily wastewater SARS-CoV-2 viral loads (population-normalized envelope copies, cube rooted) detected at (a) site B(A), (b) site E2(CBA), and (d) site G(FEDCBA) with the loads contributed by structures (d) B, (e) E, and (f) F and D estimated assuming upstream viral signals are reliably translated downstream. Red bars highlight those predictions that are negative. (g-i) The associated number of infections detected within those structures by medical services.

Supplemental Figure 8. Spearman correlations between sample locations based on SARS-CoV-2 envelope copies per liter wastewater for Fall (top half of diagram) and Spring (bottom half of diagram). The locations are clustered based on the correlation scores for the Fall 2020 semester. Significance scores of 0.05, 0.01, and 0.001 are represented by *, **, and ***, respectively.

Supplemental Figure 9. Mean values from autocorrelation function and cross-correlation functions between all sites from 0-14 days lag.


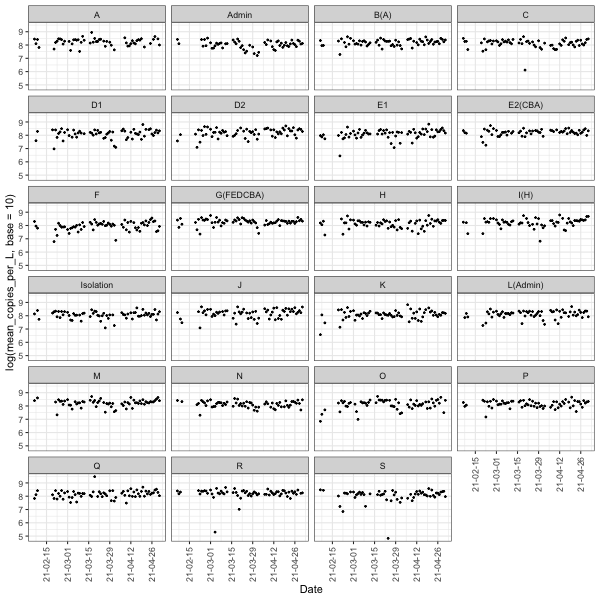


Supplemental Figure 1. Concentration (log scale) of the bovine coronavirus spike-in across all sites for all samples


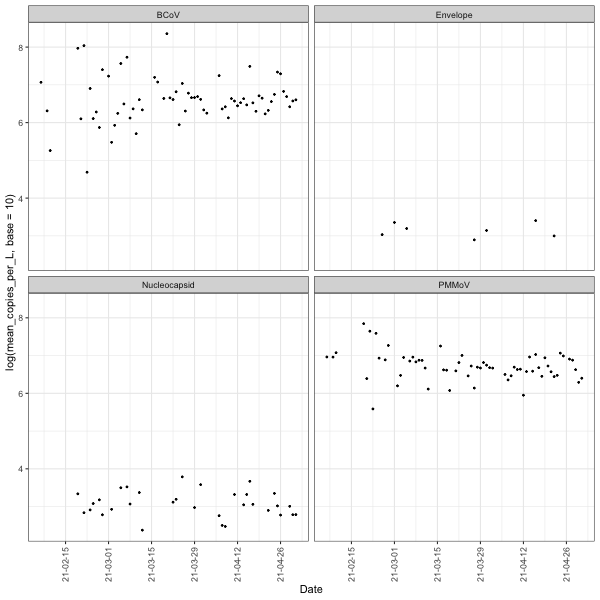


Supplemental Figure 2. Concentration (log scale) for the process blank with a bovine coronavirus recovery spike-in


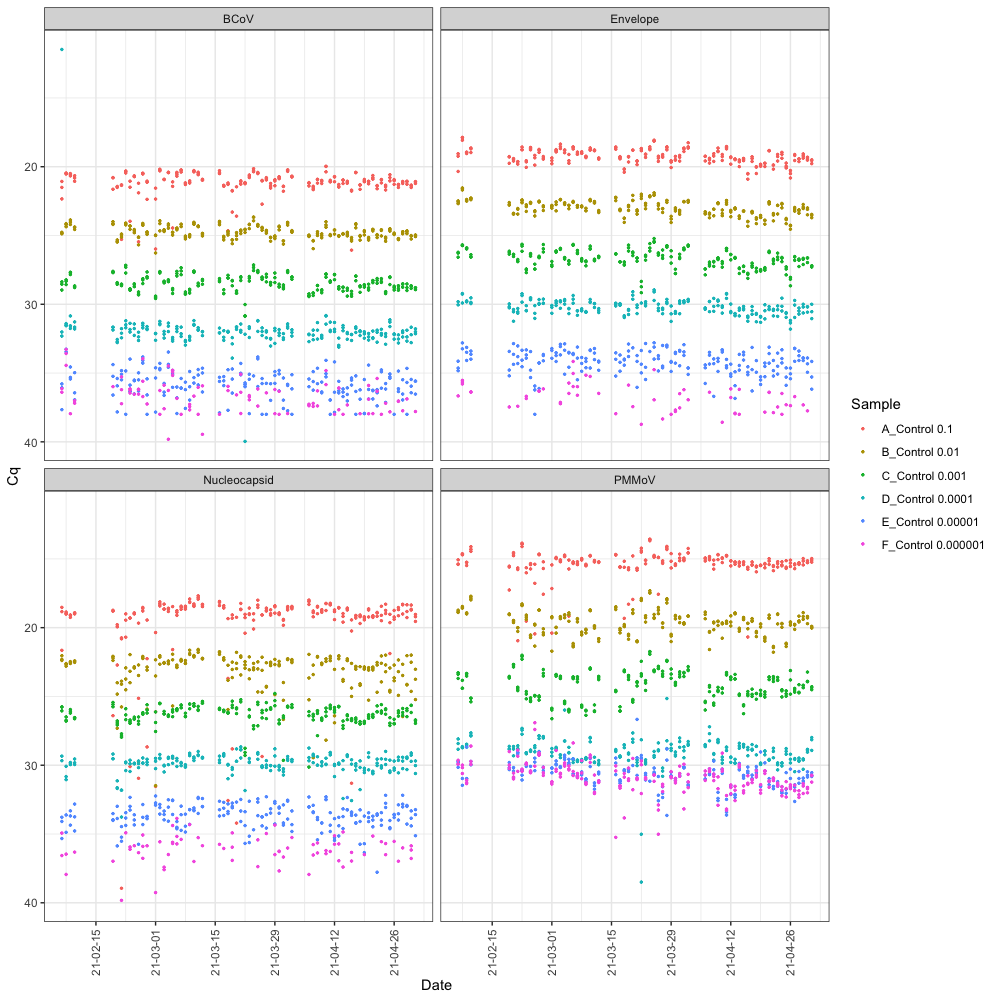


Supplemental Figure 3. SENB+ Cq values for the A-F standard curves. Standard concentrations described in Supplemental Table 10


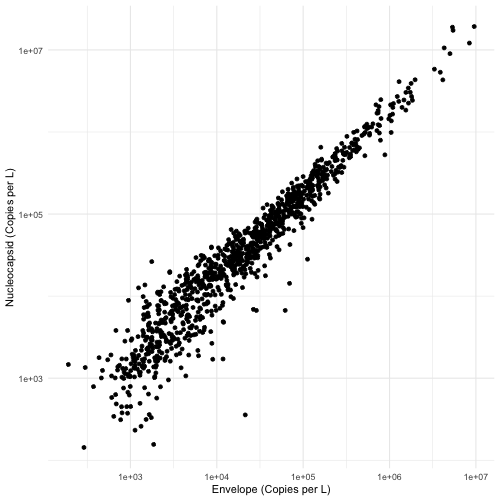


Supplemental Figure 4. Scatterplot comparing Nucleocapsid (N) and Envelope (E) copies per L wastewater across all spring samples after processing pipeline


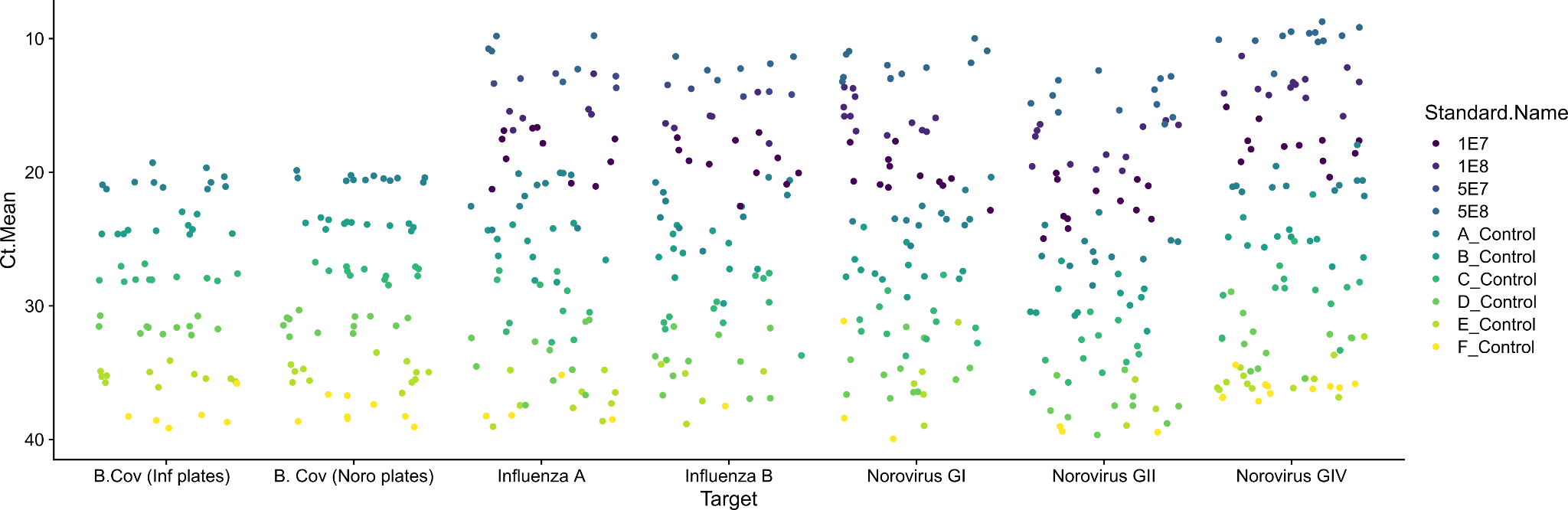


Supplemental Figure 5. Cq values for the standard curves for the norovirus and influenza assays. Standard concentrations described in Supplemental Table 10


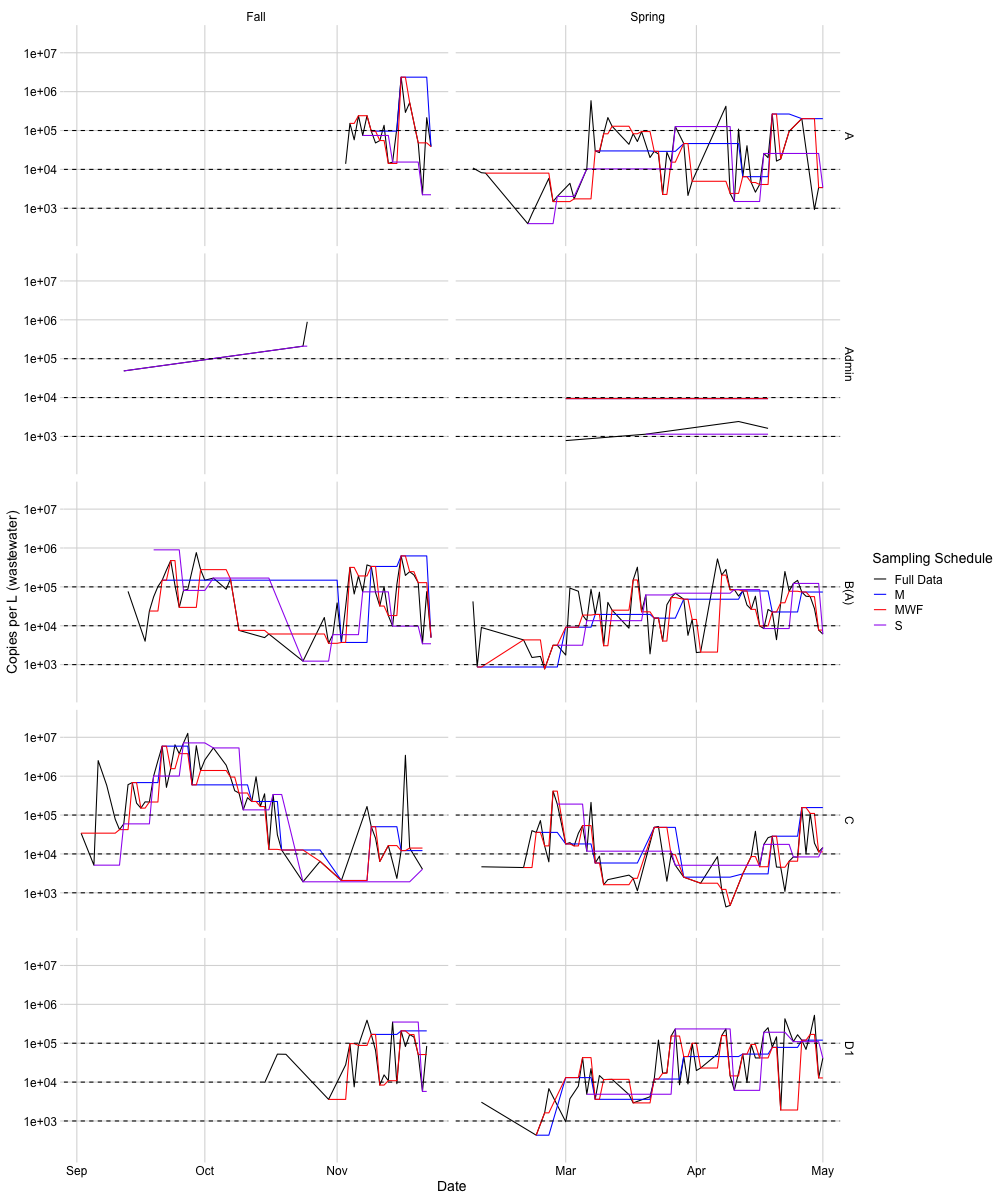


Supplemental Figure 6. Copies (Envelope) per L wastewater for full data and simulated reduced sampling schedules for all sites between fall and spring, dashed lines indicate thresholds of 1000, 10,000, and 100,000.


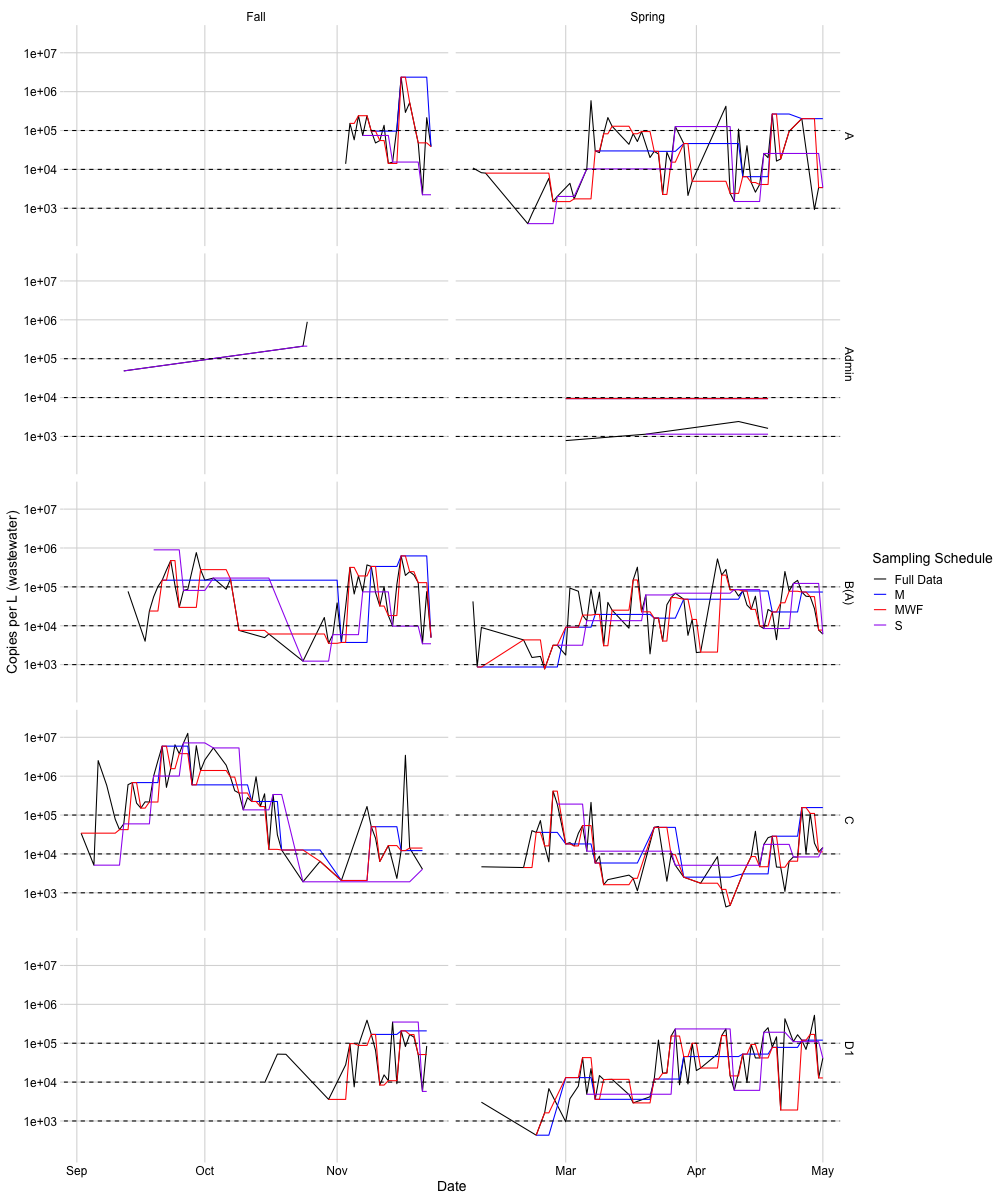


Supplemental Figure 6. [continued] Copies (Envelope) per L wastewater for full data and simulated reduced sampling schedules for all sites between fall and spring, dashed lines indicate thresholds of 1000, 10,000, and 100,000.


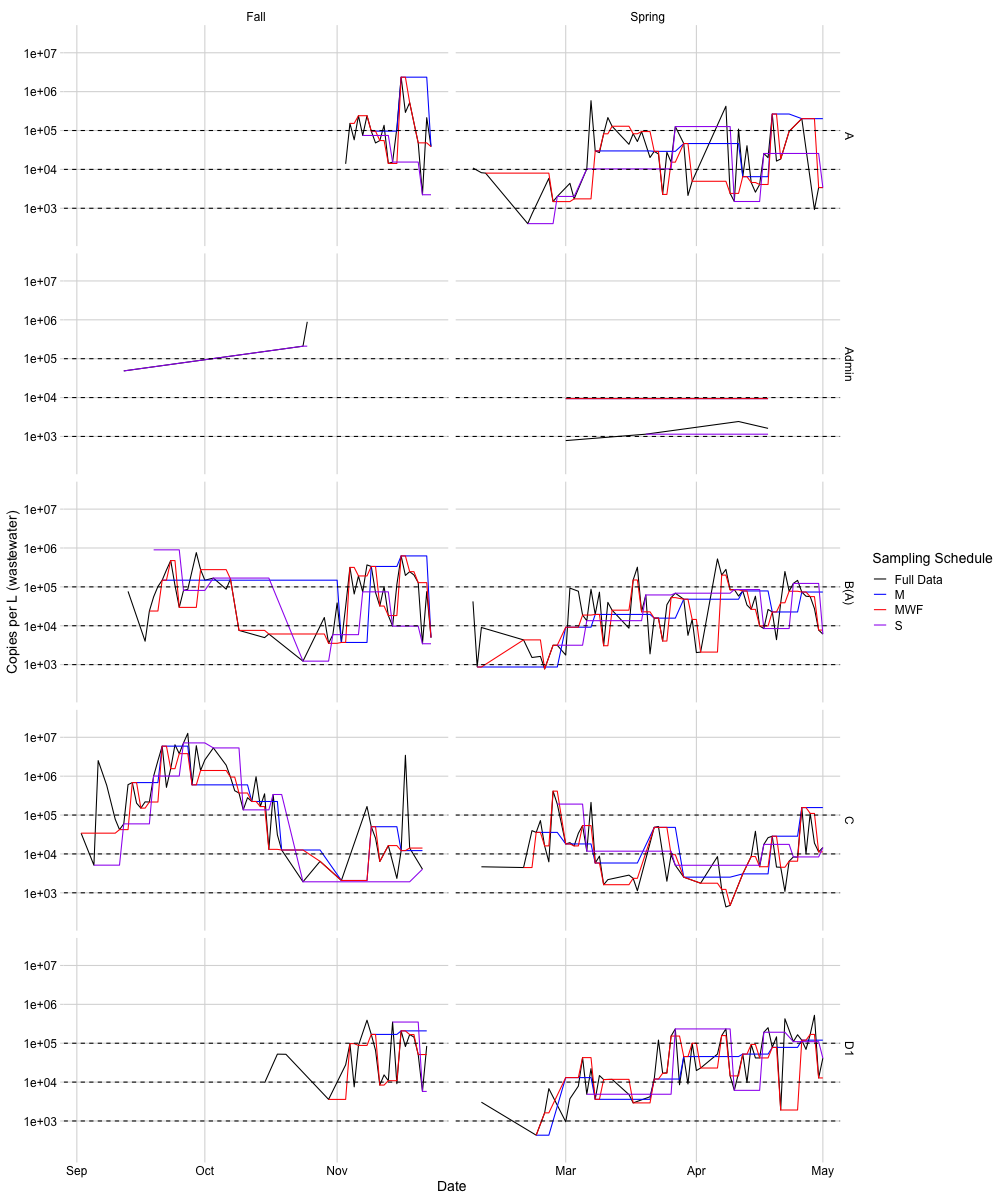


Supplemental Figure 6. [continued] Copies (Envelope) per L wastewater for full data and simulated reduced sampling schedules for all sites between fall and spring, dashed lines indicate thresholds of 1000, 10,000, and 100,000.


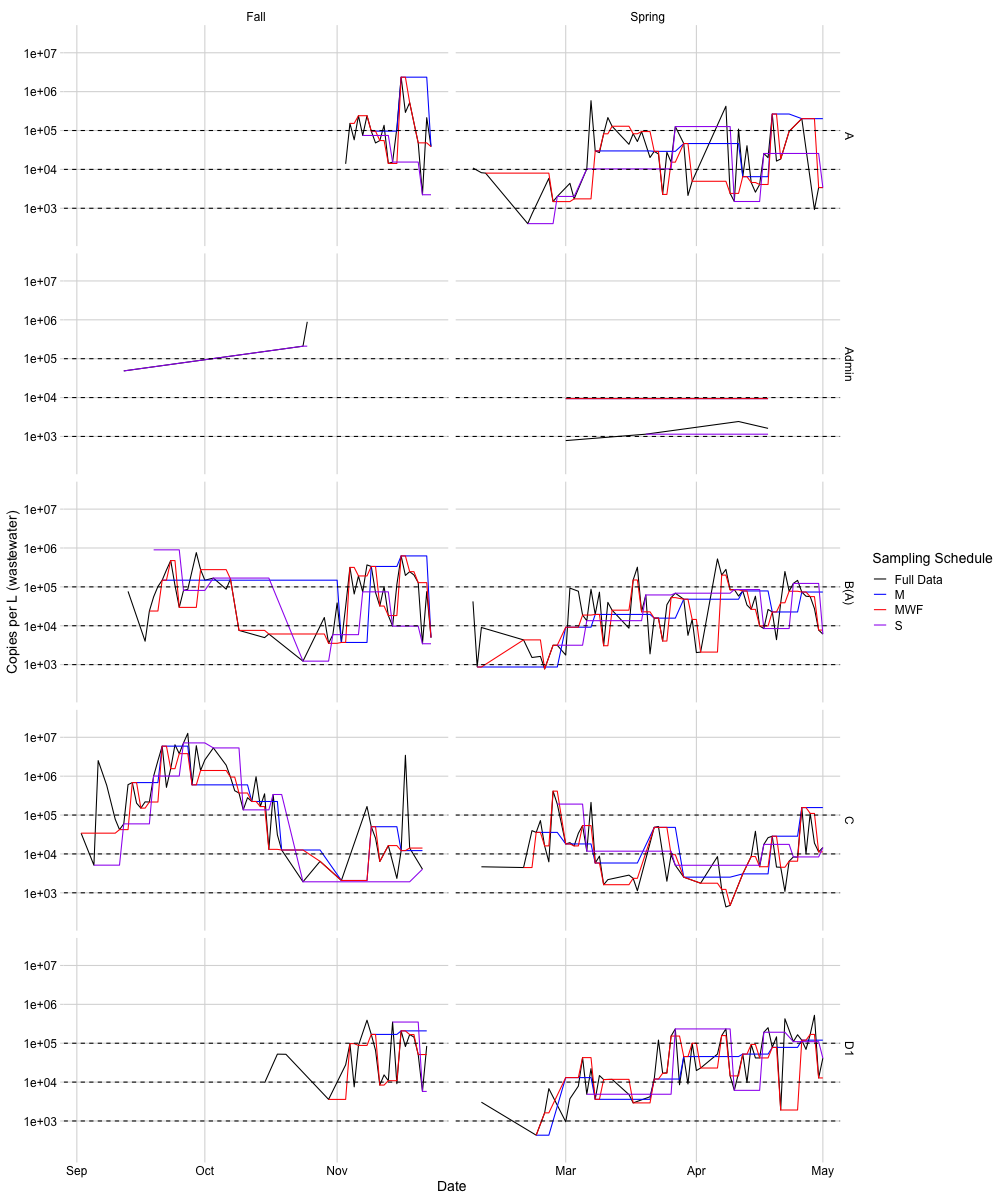


Supplemental Figure 6. [continued] Copies (Envelope) per L wastewater for full data and simulated reduced sampling schedules for all sites between fall and spring, dashed lines indicate thresholds of 1000, 10,000, and 100,000.


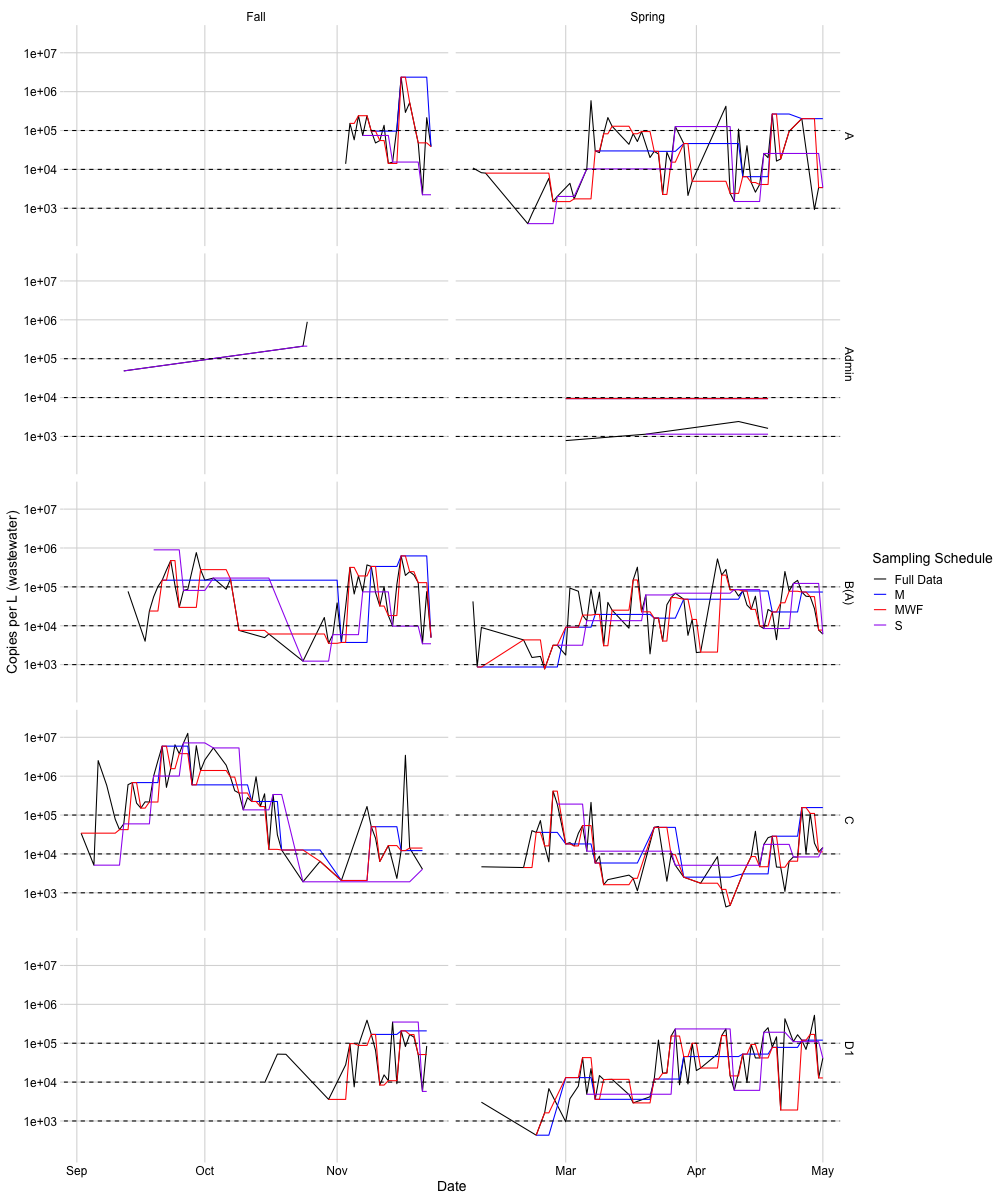


Supplemental Figure 6. [continued] Copies (Envelope) per L wastewater for full data and simulated reduced sampling schedules for all sites between fall and spring, dashed lines indicate thresholds of 1000, 10,000, and 100,000.


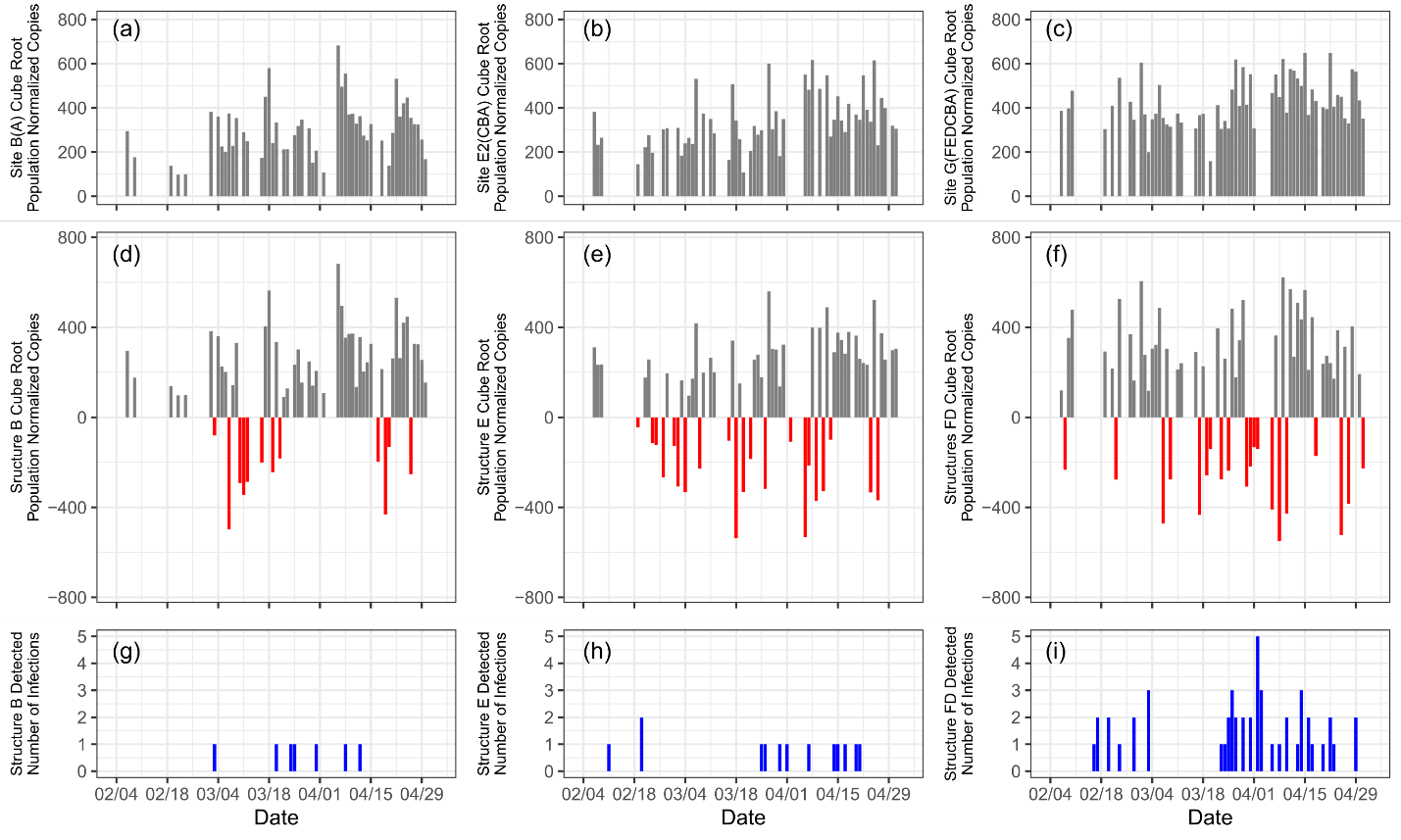


Supplemental Figure 7. Plots depict daily wastewater SARS-CoV-2 viral loads (population-normalized envelope copies, cube rooted) detected at (a) site B(A), (b) site E2(CBA), and (d) site G(FEDCBA) with the loads contributed by structures (d) B, (e) E, and (f) F and D estimated assuming upstream viral signals are reliably translated downstream. Red bars highlight those predictions that are negative. (g-i) The associated number of infections detected within those structures by medical services.


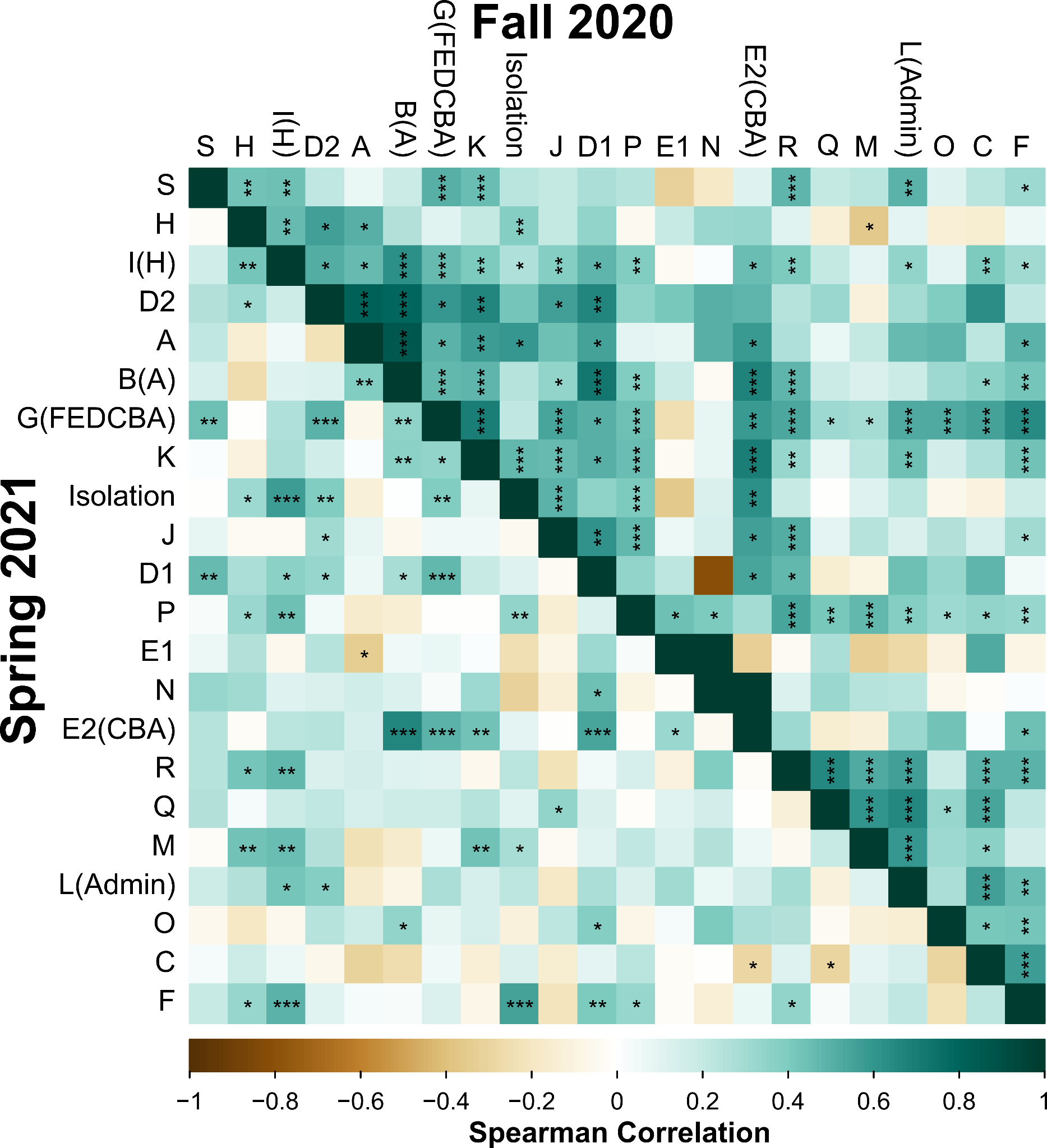


Supplemental Figure 8. Spearman correlations between sample locations based on SARS-CoV-2 envelope copies per liter wastewater for Fall (top half of diagram) and Spring (bottom half of diagram). The locations are clustered based on the correlation scores for the Fall 2020 semester. Significance scores of 0.05, 0.01, and 0.001 are represented by *, **, and ***, respectively.


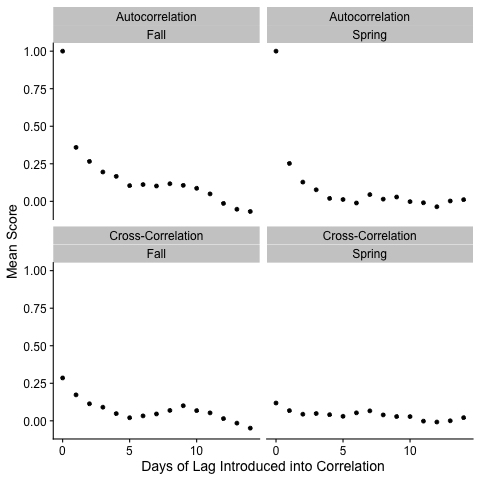


Supplemental Figure 9. Mean values from autocorrelation function and cross-correlation functions between all sites from 0-14 days lag.
