# Supplementary material for: Effectiveness of building-level sewage surveillance during both community-spread and sporadic-infection phases of SARS-CoV-2 in a university campus population
Source: FEMS Microbes. 2022 Sep 24;3:xtac024. doi: 10.1093/femsmc/xtac024 (PMC10117889; doi:10.1093/femsmc/xtac024)
Supplement: xtac024_Supplemental_Files [file xtac024_supplemental_files.zip › 20220827_Johnson_et_al_Supplemental_File_1.docx]

**Supplemental File 1. Script for processing the qPCR data in R**

---

title: "Johnson et al., Supplemental File 1"

date: "5/26/2022"

output: github_document

---

```{r setup, include=FALSE}

knitr::opts_chunk$set(echo = TRUE)

```

This file details the data cleaning procedures from “Effectiveness of building-level sewage surveillance during both community-spread and sporadic-infection phases of SARS-CoV-2 in a university campus population” by Johnson et al.

```{r}

pacman::p_load(tidyverse, lubridate, cowplot, RColorBrewer)

# loading in tables previously produced using readxl from google sheets

Cq_allstandards <- read.csv("spring_cq_supplemental_standards.txt", header = TRUE, sep = " ")

Cq_allblanks <- read.csv("spring_cq_blanks_supplemental.txt", header = TRUE, sep = " ") %>%

filter(Date != "2021-04-08" & Well.Position != "A10")

Cq_allsamples <- read.csv("spring_cq_supplemental_samples.txt", header = TRUE, sep = " ")

elution <- read.csv("elution_supplemental.txt", header = TRUE, sep = " ")

# Reformatting tables for downstream analysis

Cq_cleaning <- Cq_allstandards %>%

filter(str_detect(Sample, "Control")) %>%

filter(!is.na(Cq.Mean)) %>%

dplyr::rename(Sample.Standard = Sample) %>%

mutate(Sample.Standard = str_split(Sample.Standard, " ")) %>%

mutate(Concentration = as.numeric(map(Sample.Standard, function(x) x[2])),

Sample.Standard = as.character(map(Sample.Standard, function(x) x[1]))) %>%

unnest(c(Date, Concentration, Cq))

elution <- elution %>%

mutate(Date = as.Date(Date)) %>%

mutate(Sample = str_replace(Sample, "BLANK", "Extraction Blank"))

```

After removing triplicates which have NA values for mean, the composition of Cq means for standards is as follows

```{r, fig.width = 12}

Cq_plot <- Cq_cleaning %>%

select(Date, Target, Sample.Standard, Cq.Mean) %>%

unique()

ggplot(Cq_plot, aes(x = Target, y = Cq.Mean, color = Sample.Standard)) +

geom_jitter() + scale_color_viridis_d() + theme_cowplot() +

scale_y_reverse()

ggplot(Cq_plot, aes(sample = Cq.Mean)) + stat_qq() + stat_qq_line() + facet_grid(Target ~

Sample.Standard)

```

It is observed the distribution of Cq means for the F standard of each target closely mirrors that of the blanks.

```{r, fig.width = 12}

Cq_plot <- Cq_plot %>%

filter(Sample.Standard == "F_Control")

ggplot() + geom_jitter(data = Cq_allblanks, aes(x = Target, y = Cq)) +

geom_jitter(data = Cq_plot, aes(x = Target, y = Cq.Mean), color = "blue") +

theme_cowplot() + scale_y_reverse()

```

The high degree of overlap between points in the E standards and F standards indicate that an initial cleaning step is necessary, while the overlap between D, E, and F standards for PMMoV is likely indicative of a higher amount of contamination. The quantile-quantile plots largely seem to indicate that each standard from each target has a normal distribution of Cq values, although there may be slight deviation from normality in the envelope standards. Non-normal behavior is also observed in the Nucleocapsid A standard.

# 1. Data Correction

These data have had the following manual editing steps performed by Katie Reeves

1. Fix plate templates

a. Make sure all wells labeled correctly

b. Relabel G standard as blank

c. Switch Kitt C and Kitt W labels when appropriate

d. Switch Darley and Regent labels when appropriate

2. Omit wells:

a. That had no contents (in multicomponent plot, all fluorescence at 0)

b. That suffered from known pipetting errors (e.g., contaminated with another sample/standard)

3. Omit sample wells:

a. In which BCoV did not amplify (NOTE NOW KEEPING SINGLE AMPLIFICATIONS)

The additional processing steps were performed by William Johnson

1. Fix target thresholds

# 2. Filtering by Cq standard deviation

As triplicates with too high of a Cq variance are deemed unreliable, all standards are removed which have a standard deviation greater than an allowable threshold calculated using the poisson distribution. The same filter will be applied to sample triplicates as well.

```{r}

# producing linear models for each date/target group and calculating copy # in each standard

# using dilution level

lmw <- function(data, formula) {

broom::tidy(tryCatch(lm(data = data, formula = formula), error = function(cond) NULL))

}

Cq_lm <- function(df) {

lmw(Cq ~ log10(standard_quantity), data = df)

}

Cq_cleaning <- Cq_cleaning %>%

mutate(starting_quantity = recode(Target, PMMoV = 1.07e+08, Envelope = 1e+06,

Nucleocapsid = 1e+06, BCoV = 1e+07)) %>%

mutate(standard_quantity = starting_quantity * Concentration) %>%

group_by(Date, Target) %>%

nest() %>%

mutate(lms = map(data, Cq_lm)) %>%

unnest(data) %>%

unnest(lms) %>%

filter(term == "log10(standard_quantity)") %>%

mutate(efficiency = (10^(-1/estimate)))

# calculating the deltas between members of a triplicate

Cq_diff <- function(data) {

data %>%

mutate(rank = rank(Cq)) %>%

mutate(rank = as.character(rank)) %>%

mutate(rank = str_replace(rank, "1", "low")) %>%

mutate(rank = str_replace(rank, "2", "mid")) %>%

mutate(rank = str_replace(rank, "3", "high")) %>%

select(-Well.Position) %>%

pivot_wider(names_from = rank, values_from = Cq)

}

Cq_rank <- function(data) {

data %>%

mutate(rank = rank(Cq)) %>%

mutate(rank = as.character(rank)) %>%

mutate(rank = str_replace(rank, "1", "low")) %>%

mutate(rank = str_replace(rank, "2", "mid")) %>%

mutate(rank = str_replace(rank, "3", "high"))

}

Cq_cleaning <- Cq_cleaning %>%

group_by(Date, Target, Sample.Standard) %>%

nest() %>%

mutate(data = map(data, Cq_rank)) %>%

unnest(data)

removed_by_poisson <- Cq_cleaning %>%

group_by(Date, Target, Sample.Standard) %>%

nest() %>%

mutate(data = map(data, Cq_diff)) %>%

unnest(data) %>%

mutate(high_interval = high - mid) %>%

mutate(low_interval = mid - low) %>%

pivot_longer(cols = c(high, mid, low), names_to = "rank", values_to = "Cq")

# calculating the allowable delta for each triplicate using the poisson distribution

removed_by_poisson <- removed_by_poisson %>%

mutate(N = 10 * efficiency^(35 - Cq.Mean)) %>%

mutate(nlow = 0.5 * qchisq(0.025, 2 * N)) %>%

mutate(nhigh = 0.5 * qchisq(0.975, 2 * N + 2)) %>%

mutate(delta = (log(nhigh) - log(nlow))/log(efficiency)) %>%

mutate(delta = ifelse(delta < 0.5, 0.5, delta)) %>%

mutate(delta = ifelse(delta > 2, 2, delta))

# creating a table of amplifications where:

# highest amplification is removed if distance between high & mid is > allowable delta

# mid amplification is removed if only one (but not both) of high/low remain

# & the distance from mid to that value > allowable delta

# or if both high/low remain & distance from mid to both > allowable delta

# lowest amplification is removed if distance between low & mid is > allowable delta

# for visual representation uncomment code below

# pacman::p_load(lobstr)

# ast(filter((high_interval > delta & rank == "high") | ((high_interval >

# delta | is.na(high_interval)) & (low_interval > delta | is.na(low_interval)) &

# !(is.na(high_interval) & is.na(low_interval)) & rank == "mid") |

# (low_interval > delta & rank == "low")))

removed_by_poisson <- removed_by_poisson %>%

filter((high_interval > delta & rank == "high") | ((high_interval >

delta | is.na(high_interval)) & (low_interval > delta | is.na(low_interval)) &

!(is.na(high_interval) & is.na(low_interval)) & rank == "mid") |

(low_interval > delta & rank == "low")) %>%

select(Date, Target, Sample.Standard, rank, delta)

# removing the amplifications calculated in the last step

Cq_cleaning <- Cq_cleaning %>%

anti_join(removed_by_poisson, by = c("Date", "Target", "Sample.Standard",

"rank")) %>%

group_by(Date, Target, Sample.Standard) %>%

mutate(Cq.Mean = mean(Cq, na.rm = TRUE)) %>%

ungroup()

```

```{r, fig.width = 12}

Cq_plot <- Cq_cleaning %>%

select(Date, Target, Sample.Standard, Cq.Mean) %>%

unique()

ggplot(Cq_plot, aes(x = Target, y = Cq.Mean, color = Sample.Standard)) +

geom_jitter() + scale_color_viridis_d() + theme_cowplot() + scale_y_reverse()

ggplot(Cq_plot, aes(sample = Cq.Mean)) + stat_qq() + stat_qq_line() + facet_grid(Target ~

Sample.Standard)

```

This filter seems to have removed the non normal behavior from the nucleocapsid A standard.

# 3. Blank Comparison

In order to eliminate any standards which are below the LOQ (LOD?) for a given run, a comparison is made between the standards and blanks for each run. When a single blank replicate has a lower Cq value for a target than a single standard, that standard is removed from the run.

First the lowest Cq blank for each run is determined, then that value is subtracted from each standard CT. If positive, the standard is kept; if negative, it is removed.

```{r}

# creating a table with the lowest cq value for a blank in each date/target group

blank_mins <- Cq_allblanks %>%

group_by(Date, Target) %>%

filter(!is.na(Cq), Sample == "Blank") %>%

filter(Cq == min(Cq)) %>%

arrange(Date, Target) %>%

select(-Sample)

# repeating above operation for extraction blanks

extraction_blank_mins <- Cq_allblanks %>%

group_by(Date, Target) %>%

filter(!is.na(Cq), Sample == "Extraction Blank") %>%

filter(Cq == min(Cq)) %>%

arrange(Date, Target) %>%

select(-Sample)

# finding all date/target/standard groups which had an amplification below the associated minimum blank

standards_below_blank <- Cq_cleaning %>%

full_join(blank_mins, by = c("Date", "Target"), suffix = c(".Standard",

".Blank")) %>%

mutate(blankminstandard = Cq.Blank - Cq.Standard) %>%

filter(!is.na(blankminstandard)) %>%

group_by(Date, Target, Sample.Standard) %>%

filter(blankminstandard == min(blankminstandard)) %>%

arrange(Date, Target) %>%

filter(blankminstandard < 0)

# removing the groups found in the last step

Cq_cleaning <- Cq_cleaning %>%

anti_join(standards_below_blank, by = c("Date", "Target", "Sample.Standard"))

```

The following triplicates were removed from the data

```{r, fig.width = 12}

ggplot(standards_below_blank, aes(x = Target))+

geom_bar() +

theme_cowplot()

```

To produce this distribution of means

```{r, fig.width = 12}

Cq_plot <- Cq_cleaning %>%

select(Date, Target, Sample.Standard, Cq.Mean) %>%

unique()

ggplot(Cq_plot, aes(x = Target, y = Cq.Mean, color = Sample.Standard)) +

geom_jitter() +

scale_color_viridis_d() + theme_cowplot() +

scale_y_reverse()

```

A bit better!

Now let's remove all non-BCoV standards with a Cq value higher than the extraction blanks

```{r}

# repeating above operation for extraction blanks

standards_below_extraction_blank <- Cq_cleaning %>%

full_join(extraction_blank_mins, by = c("Date", "Target"), suffix = c(".Standard",

".EBlank")) %>%

mutate(eblankminstandard = Cq.EBlank - Cq.Standard) %>%

filter(!is.na(eblankminstandard)) %>%

group_by(Date, Target, Sample.Standard) %>%

filter(eblankminstandard == min(eblankminstandard)) %>%

arrange(Date, Target) %>%

filter(eblankminstandard < 0, Target != "BCoV")

Cq_cleaning <- Cq_cleaning %>%

anti_join(standards_below_extraction_blank, by = c("Date", "Target",

"Sample.Standard"))

# processing blank mins to set LoD in later sample steps

LoD <- Cq_cleaning %>%

group_by(Date, Target) %>%

mutate(LoD = min(standard_quantity)) %>%

ungroup() %>%

select(Date, Target, LoD) %>%

mutate(Date = as.Date(Date, format = "%Y-%m-%d")) %>%

unique()

blank_mins <- blank_mins %>%

select(Date, Target, Cq)

all_blank_mins <- extraction_blank_mins %>%

select(Date, Target, Cq) %>%

full_join(blank_mins, by = c("Date", "Target"), suffix = c(".extraction",

".blank")) %>%

mutate(lowest_blank = pmin(Cq.extraction, Cq.blank, na.rm = TRUE)) %>%

mutate(lowest_blank = ifelse(Target == "BCoV", Cq.blank, lowest_blank)) %>%

select(-Cq.extraction, -Cq.blank) %>%

mutate(Date = as.Date(Date))

```

```{r, fig.width = 12}

ggplot(standards_below_extraction_blank, aes(x = Target)) + geom_bar() +

facet_wrap(~Sample.Standard) + theme_cowplot()

```

```{r, fig.width = 10}

Cq_plot <- Cq_cleaning %>%

select(Date, Target, Sample.Standard, Cq.Mean) %>%

unique()

ggplot(Cq_plot, aes(x = Target, y = Cq.Mean, color = Sample.Standard)) +

geom_jitter() + scale_color_viridis_d() + theme_cowplot() + scale_y_reverse()

```

# 4. Cq intervals

The following code plots the distribution of the daily Cq intervals between standards for each target.

```{r}

Cq_intervals <- Cq_cleaning %>%

select(Date, Target, Cq.Mean, Sample.Standard) %>%

unique() %>%

pivot_wider(names_from = Sample.Standard, values_from = Cq.Mean) %>%

mutate(

"A -> B" = B_Control - A_Control,

"B -> C" = C_Control - B_Control,

"C -> D" = D_Control - C_Control,

"D -> E" = E_Control - D_Control,

"E -> F" = F_Control - E_Control) %>%

select(-contains("_Control")) %>%

pivot_longer(cols = contains("->"), names_to = "Interval", values_to = "Cq.Mean")

```

```{r, fig.width = 12}

ggplot(data = Cq_intervals, aes(x = Target, y = Cq.Mean)) + geom_violin() +

facet_wrap(~Interval) + theme_cowplot()

```

There is a clear pattern of bunching in the lower Cq intervals, indicating that they are below the limit of quantification. Defining the LoQ as the region of the standard curve which is linear, we will construct a linear regression using the A, B, and C standard for each target, and eliminate the D, E, and F standards which lie outside of the 95% prediction interval of that regression. If the mean of a D, E, or F triplicate falls outside of that interval, it is deemed to be beyond the limit of detection.

```{r}

# creating lm and prediction interval for each the A, B, and C standard from each target

lmw_untidy <- function(data, formula) {

tryCatch(lm(data = data, formula = formula), error = function(cond) NULL)

}

Cq_lm_untidy <- function(data1, data2) {

lmw_untidy(Cq ~ log10(standard_quantity), data = data1)

}

predicts <- function(object, newdata) {

as_tibble(tryCatch(predict(object = object, newdata, interval = "prediction",

level = 0.95), error = function(cond) NULL))

}

Cq_LoQ <- Cq_cleaning %>%

select(Date, Target, Sample.Standard, Cq, Cq.Mean, Concentration, standard_quantity) %>%

filter(Sample.Standard %in% c("A_Control", "B_Control", "C_Control")) %>%

group_by(Target) %>%

nest() %>%

dplyr::rename(ABC = data)

Cq_predictions <- Cq_cleaning %>%

select(Target, Sample.Standard, standard_quantity) %>%

unique() %>%

filter(Sample.Standard %in% c("D_Control", "E_Control", "F_Control")) %>%

group_by(Target, Sample.Standard) %>%

nest() %>%

dplyr::rename(DEF = data) %>%

full_join(Cq_LoQ, by = c("Target")) %>%

mutate(lms = map(ABC, Cq_lm_untidy)) %>%

mutate(predictions = map2(lms, DEF, predicts)) %>%

unnest(DEF) %>%

unnest(predictions) %>%

select(Target, Sample.Standard, fit, upr, lwr) %>%

full_join(Cq_cleaning, by = c("Target", "Sample.Standard"))

# removing D, E, and F standards which lie outside of the prediction interval

Cq_outofrange <- Cq_predictions %>%

filter(Cq.Mean < lwr | Cq.Mean > upr)

Cq_cleaning <- Cq_predictions %>%

filter(Cq.Mean >= lwr & Cq.Mean <= upr | Sample.Standard %in% c("A_Control",

"B_Control", "C_Control"))

```

The following plot display the upper and lower bounds of the prediction interval for standards D, E, and F, as well as the distribution of mean Cqs

```{r, fig.width = 12}

Cq_bounds <- Cq_predictions %>%

select(lwr, upr, Target, Sample.Standard, standard_quantity) %>%

unique()

ggplot() + geom_point(data = Cq_predictions, aes(x = log10(standard_quantity),

y = Cq.Mean)) + geom_line(data = Cq_bounds, aes(y = lwr, x = log10(standard_quantity))) +

geom_line(data = Cq_bounds, aes(y = upr, x = log10(standard_quantity))) +

facet_wrap(~Target) + scale_x_reverse() + scale_y_reverse() + theme_cowplot()

```

These triplicates were removed from the data

```{r, fig.width = 12}

ggplot(data = Cq_outofrange, aes(x = Target)) + geom_bar() + facet_wrap(~Sample.Standard) +

theme_cowplot()

```

Which produce these intervals

```{r}

Cq_intervals <- Cq_cleaning %>%

select(Date, Target, Cq.Mean, Sample.Standard) %>%

unique() %>%

pivot_wider(names_from = Sample.Standard, values_from = Cq.Mean) %>%

mutate(

"A -> B" = B_Control - A_Control,

"B -> C" = C_Control - B_Control,

"C -> D" = D_Control - C_Control,

"D -> E" = E_Control - D_Control,

"E -> F" = F_Control - E_Control) %>%

select(-contains("_Control")) %>%

pivot_longer(cols = contains("->"), names_to = "Interval", values_to = "Cq.Mean")

```

```{r, fig.width = 12}

ggplot(data = Cq_intervals, aes(x = Target, y = Cq.Mean)) + geom_violin() +

facet_wrap(~Interval) + theme_cowplot()

```

```{r, fig.width = 10}

Cq_plot <- Cq_cleaning %>%

select(Date, Target, Sample.Standard, Cq.Mean) %>%

unique()

ggplot(Cq_plot, aes(x = Target, y = Cq.Mean, color = Sample.Standard)) +

geom_jitter() + scale_color_viridis_d() + theme_cowplot() + scale_y_reverse()

```

Before calculating the slope and efficiency for each day and standard, it is necessary to remove those standard curves which have less than three levels remaining, as anything less is likely to be unreliable.

```{r}

# calculating the remaining standard levels for each date/target group

Cq_tally <- Cq_cleaning %>%

group_by(Date, Target) %>%

distinct(Sample.Standard) %>%

tally() %>%

full_join(Cq_cleaning)

# removing date/target groups with less than 3 remaining levels

n_less_than_3 <- Cq_tally %>%

filter(n < 3)

Cq_cleaning <- Cq_tally %>%

filter(n >= 3)

```

Now that the data is cleaned up, we'll need to recalculate the slope and efficiency for each standard.

Note: Starting quantities are ten fold higher than A control quantities, because A has a concentration of 0.1

```{r}

# shifts intercept and slope to be on the same line

widen_models <- function(data) {

data %>%

select(term, estimate) %>%

pivot_wider(names_from = term, values_from = estimate)

}

# calculating lms

Cq_models <- Cq_cleaning %>%

mutate(starting_quantity = recode(Target, PMMoV = 1.07e+08, Envelope = 1e+06,

Nucleocapsid = 1e+06, BCoV = 1e+07)) %>%

mutate(standard_quantity = starting_quantity * Concentration) %>%

filter(Sample.Standard != "F_Control" | Date != "2021-02-25") %>%

group_by(Date, Target) %>%

nest() %>%

mutate(lms = map(data, Cq_lm)) %>%

mutate(lms = map(lms, widen_models)) %>%

unnest(data) %>%

unnest(lms) %>%

dplyr::rename(c(Intercept = `(Intercept)`, Slope = `log10(standard_quantity)`)) %>%

mutate(efficiency = (-1 + 10^(-1/Slope)) * 100)

```

```{r, fig.width = 12}

Cq_plot <- Cq_models %>%

select(Date, Target, Sample.Standard, Cq.Mean, Slope, efficiency) %>%

unique()

ggplot(Cq_plot, aes(x = Target, y = Slope, color = Sample.Standard)) +

geom_jitter() + scale_color_viridis_d() + theme_cowplot() + scale_y_reverse()

ggplot(Cq_plot, aes(x = Target, y = efficiency, color = Sample.Standard)) +

geom_jitter() + scale_color_viridis_d() + theme_cowplot()

```

# 5. Cleaning sample data

The linear regressions calculated from the cleaned standards now need to be joined with the associated sample data, but first some cleaning steps must be applied to the sample data.

```{r}

# applying steps from #2 to sample data

Cq_models <- Cq_models %>%

group_by(Date, Target) %>%

mutate(LoQ = min(standard_quantity)) %>%

select(Date, Target, Slope, Intercept, LoQ) %>%

unique()

Cq_cleaning_samples <- Cq_allsamples %>%

full_join(Cq_models, by = c("Date", "Target")) %>%

filter(!is.na(Cq.Mean)) %>%

mutate(Date = as.Date(Date)) %>%

mutate(efficiency = (10^(-1/Slope)))

```

Let's take a look at the Cq Means from the sample data.

```{r, fig.width = 15, fig.height = 20}

Cq_plot <- Cq_cleaning_samples %>%

select(Date, Target, Cq.Mean, Sample) %>%

unique()

color_count <- length(unique(Cq_plot$Sample))

Dark2_Ramp <- colorRampPalette(brewer.pal(8, "Dark2"))

ggplot(Cq_plot, aes(x = Date, y = Cq.Mean, color = Sample)) + geom_jitter(size = 3) +

theme_cowplot() + scale_color_manual(values = Dark2_Ramp(color_count)) +

facet_wrap(~Target, ncol = 1) + scale_y_reverse() + scale_x_date(date_break = "1 month") +

guides(color = guide_legend(ncol = 1))

```

```{r}

Cq_cleaning_samples <- Cq_cleaning_samples %>%

group_by(Date, Target, Sample) %>%

nest() %>%

mutate(data = map(data, Cq_rank)) %>%

unnest(data)

Cq_ranked_samples <- Cq_cleaning_samples %>%

group_by(Date, Target, Sample) %>%

nest() %>%

mutate(data = map(data, Cq_diff)) %>%

unnest(data) %>%

mutate(high_interval = high - mid) %>%

mutate(low_interval = mid - low) %>%

pivot_longer(cols = c(high, mid, low), names_to = "rank", values_to = "Cq")

# calculating allowable delta from poisson distribution

Cq_poisson_samples <- Cq_ranked_samples %>%

mutate(N = 10 * efficiency^(35 - Cq.Mean)) %>%

mutate(nlow = 0.5 * qchisq(0.025, 2 * N)) %>%

mutate(nhigh = 0.5 * qchisq(0.975, 2 * N + 2)) %>%

mutate(delta = (log(nhigh) - log(nlow))/log(efficiency)) %>%

mutate(delta = ifelse(delta < 0.5, 0.5, delta)) %>%

mutate(delta = ifelse(delta > 2, 2, delta))

# creating list of amplifications which fail poisson filter

removed_by_poisson <- Cq_poisson_samples %>%

filter((high_interval > delta & rank == "high") | (((is.na(high_interval) |

is.na(low_interval)) & (!is.na(high_interval) | !is.na(low_interval)) &

(high_interval > delta | low_interval > delta) | (!is.na(high_interval) &

!is.na(low_interval)) & (high_interval > delta & low_interval >

delta)) & rank == "mid") | (low_interval > delta & rank == "low")) %>%

select(Date, Target, Sample, rank, delta)

# remove amplifications identified in the last step

Cq_cleaning_samples <- Cq_cleaning_samples %>%

anti_join(removed_by_poisson, by = c("Date", "Target", "Sample", "rank")) %>%

group_by(Date, Target, Sample) %>%

mutate(Cq.Mean = mean(Cq, na.rm = TRUE)) %>%

ungroup()

```

The following samples were removed by the filter

```{r, fig.width = 12}

ggplot(removed_by_poisson, aes(x = Target)) +

geom_bar() + theme_cowplot()

```

```{r}

# caclulates number of copies in each reaction from efficiency, intercept, & cq

# calculates the number of copies per liter from processing metrics and

# the number of copies in each reaction volume

calc_cp_per_L <- function(quantity, volume_concentrate, volume_processed) {

(quantity/5) * (50/230) * (volume_concentrate/volume_processed)

}

Cq_copies_per_L <- Cq_cleaning_samples %>%

mutate(quantity = efficiency^((Intercept - Cq))) %>%

left_join(elution, by = c("Date", "Sample")) %>%

mutate(volume_concentrate_microL = (as.numeric(Weight.Eluent.Collected..g.)) *

1000) %>%

mutate(volume_processed_L = (as.numeric(Weight.Sample.Eluted..g.))/1000) %>%

mutate(copies_per_L = pmap(list(quantity, volume_concentrate_microL,

volume_processed_L), calc_cp_per_L)) %>%

mutate(copies_per_L = as.numeric(copies_per_L)) %>%

group_by(Date, Target, Sample) %>%

mutate(mean_copies = mean(quantity, na.rm = TRUE)) %>%

mutate(Above_LoQ = ifelse(mean_copies > LoQ, TRUE, FALSE)) %>%

mutate(mean_copies_per_L = mean(copies_per_L, na.rm = TRUE)) %>%

select(Date, Target, Sample, Cq, Cq.Mean, mean_copies, mean_copies_per_L,

Slope, Above_LoQ, LoQ, volume_concentrate_microL, volume_processed_L)

```

```{r, warning = FALSE}

samples_below_blank <- Cq_copies_per_L %>%

full_join(all_blank_mins, by = c("Date", "Target")) %>%

mutate(blankminstandard = lowest_blank - Cq) %>%

group_by(Date, Target, Sample) %>%

summarise(blankminstandard = min(blankminstandard, na.rm = TRUE)) %>%

mutate(blankminstandard = ifelse(is.infinite(blankminstandard), NA, blankminstandard)) %>%

arrange(Date, Target) %>%

mutate(Above_LoD = ifelse(is.na(blankminstandard) | blankminstandard > 0, TRUE, FALSE))

Cq_copies_per_L <- Cq_copies_per_L %>%

select(-Cq) %>%

full_join(samples_below_blank, by = (c("Date", "Target", "Sample"))) %>%

unique() %>%

full_join(LoD, by = c("Date", "Target")) %>%

unique() %>%

mutate(LoD_per_L = pmap(list(LoD, volume_concentrate_microL,

volume_processed_L), calc_cp_per_L)) %>%

mutate(LoD_per_L = as.numeric(LoD_per_L)) %>%

mutate(contains_values = TRUE)

```

```{r, fig.width = 10}

Cq_plot <- Cq_copies_per_L

ggplot(Cq_plot, aes(x = Above_LoQ)) +

geom_bar() + facet_wrap(~ Target) + theme_cowplot()

ggplot(Cq_plot, aes(x = Above_LoQ, y = Cq.Mean)) +

geom_violin() + facet_wrap(~ Target) + theme_cowplot() +

scale_y_reverse()

```

```{r}

Cq_cleaned_samples <- Cq_allsamples %>%

select(Date, Target, Sample) %>%

mutate(Date = as.Date(Date)) %>%

unique() %>%

full_join(Cq_copies_per_L, by = c("Date", "Target", "Sample")) %>%

mutate(contains_values = ifelse(is.na(contains_values), FALSE, contains_values))

write.table(Cq_cleaned_samples, "copies_per_L.txt")

checking <- read.table("copies_per_L.txt")

```
