# Supplementary material for: Effectiveness of building-level sewage surveillance during both community-spread and sporadic-infection phases of SARS-CoV-2 in a university campus population
Source: FEMS Microbes. 2022 Sep 24;3:xtac024. doi: 10.1093/femsmc/xtac024 (PMC10117889; doi:10.1093/femsmc/xtac024)
Supplement: xtac024_Supplemental_Files [file FEMSMC-2022-011 supplementary data - reviews and author response.pdf › FEMSMC-2022-011 supplementary data - reviews and author response.pdf]

Reviews of manuscript FEMSMC-2022-011

Reviewer 1: Dr Merel Bot, UCLA
Recommendation: Minor Revision

Very meaningful work! These are questions that live amongst people in the wastewater surveillance world, and it is great to have some of those questions explored.
The text is however sometimes a little hard to follow. More straight to the point sentences would make it more reader-friendly. An example; Change sentences like "The null hypothesis that there is no difference between semesters in frequency
of inaccurate calls was rejected for a Fisher's exact test (p-value = 2.2x10-16)." to something short and informative like "Frequency of inaccurate calls was significantly different between semesters (Fisher's exact test, p-value = 2.2x10-16)." Ending paragraphs with a one sentence conclusion would also be helpful, as right now many parts need to be read multiple times to understand what's being said instead of immediately understanding the take home message. For example: "Taken together, these data indicate that the higher caseload and resulting concentrations of SARS-CoV-2 within wastewater experienced during the Fall semester increase the absolute value of the error between the subsampled and the actual recorded daily value, but not necessarily the normalized error." So what does that mean? What is the take away exactly? This conclusion is only easy to understand when having read the paper sentence by sentence in depth, instead of it providing a short conclusion and the significance/impact of such a finding.
Figures are also hard to interpret, and I am unsure I am understanding all of it. For example in figure 2; as measured values are shaded in black according to the legend, which I am not sure where I can see that (the black line?). Then it says associated lines for the reduced sampling series, but then refers to colors (which are not really lines, more shaded areas?). Not sure how informative this figure is, as to me it is more confusing than explanatory.
Figure 3 is also hard to read. What is the take home message here? Maybe just show and example from one building instead of multiple, and move the others to the supplement. That way the resolution is more clear and maybe the graphs are easier to understand. Point with arrows to areas the reader should pay attention to maybe? It is a very meaningful exploration and definitely a question in the field, the take home message is just hard to understand from looking at the figure.
Figure 4 and 5 seem like they would be better suited for the supplemental section, with a short description in the text of the findings. These findings need additional work to really be meaningful and sound, since there are a lot of limitations and unmeasured covariates to this data, making it hard to draw conclusions from.
Overall great work, appreciate the exploration of flu and norovirus.

Reviewer 2: Dr Paul Monis
Recommendation: Minor Revision

This is an interesting and well-written paper. I only have some minor queries or suggestions:

- I understand the reason for citing methods used from an earlier paper, but as a reader of papers I find it frustrating to have to go look up a different paper. It would be better to provide a precis of the method or to provide the details in the supp info

- It is not completely clear to me what QC was done, especially to control for lab contamination. This can be especially important in a low prevalence context when most samples are negative / low positive and only one or a few samples are strong positives. Were extraction sample blanks / extraction blanks run, if yes, what frequency?

-in terms of assay performance, how did the two SARS assays compare in terms of virus numbers detected (E vs N)?

- have other fecal indicators been considered? While PMMoV is a fecal indicator, I am not sure how useful it is as a universal marker for normalizing sewage strength given that it is derived from diet, rather than being part of the human gut flora. While FRNA meet this criterion, they are highly variable. Data I have

seen suggests fecal indicators such as Bacteroides are more stable and also at higher numbers compared with FRNA

- I am not sure if the authors have considered the variability in virus excretion between COVID cases. Depending on the review, something like only 40-50% of cases excrete SARS-CoV-2 in feces, so this could also explain variability in virus numbers when examining virus detection from individual locations in series (A, B, C etc)

- Have the authors considered if different strains result in different virus excretion patterns? The data from SARS WBE that I have been involved in suggests that delta (probably also omicron) are shed at levels orders of magnitude higher than the earlier variants

- Figure 2 mentions black shading, do you mean the black line in the graph?

Reviewer 3: Matthew J. Wade
Recommendation: Major Revision

This was a comprehensive and interesting study on the utility of WBE for monitoring disease (principally, COVID-19) on a campus in the US. I have made extensive comments and suggestions below, but in summary, the following areas need improvement:

- Clarity, especially in description of methods (particularly stats/numeric/relational) and when making overarching statements (of purported fact)
- The style of writing is generally good, but does become informal in places. I did not point these out, but would recommend having an independent and qualified party review the language in the manuscript.
- The supplementary material, i.e. the data, is best delivered as data files rather than copied into unusuable format (i.e. text processing document).

The manuscript touches on a lot of questions that are under discussion related to the value of WBE for public health - it is good that you comment on some of the uncertainty, and complexities, in your study and utility of WBE generally. I think the manuscript is dense with information, mostly valuable, if not entirely unique (many similar studies out there, but this at least approaches from a fairly novel angle, if not always successfully). I would consider publication after major revision.

L43: "scale with the objectives" -> should be scale with the importance or level of risk/impact to public health – or just 'level of surveillance is objective dependent'? Objectives are not quantifiable in the general sense.
L48-50: This depends. Wastewater monitoring specifically to inform public health action has precedents, but it has not been 'widely applied'. While you could argue that WBE during COVID has seen wider application, it is certainly not standardised where it is implemented. This forms one of the fundamental 'next steps' for WBE in my opinion – this includes the end to end SOPs/protocols/processes.
L59: At least one of these references (Sweetapple et al) is non-US campus WBE. Please rephrase, e.g. "Across the United States and elsewhere" (L55). Do you have data on number of campuses in total (across US at least), and possible population covered?
L70: Not sure 'robust' is the correct word here. 'significant/contiguous' ?
L76-78: I am not convinced this provides the insight you suggest. It provides knowledge of local scale utility of WBE, I'm not sure how it provides national indication of progression. Please elaborate.
L81: do you mean 'social network', could be rephrased as looks like a missing word after 'social' – societal behaviours?
L86: Font size changes
L83-88: A bit confusing. You seem to frame the argument around 'individualized testing' but do not mention how this factors into your sewer sampling strategy. Or does it not matter?
L94-95: This is a spurious statement. The article you cite [Xie et al] discusses chemical marker usage and its impact on WBE data (i.e. for normalization), but your statement implied the more complex social interactions and behaviours have been researched and I would expect to see some social science/epidemiology papers discussing this. Although I am aware of some studies, I don't think the true impact of mobility, demographics and bathroom habits have been studied systematically – some work on bathroom habits on air transport/terminals has been performed [in preparation, not published], but at larger scale, I'm not sure. Please elaborate or temper the statement.

L98: why is it key? What is the motivator for WBE when flue and norovirus surveillance clinically appears to be generally effective…what about enterovirus, polio – which have precedent, but are still of concern [given recent news].
L109: you discuss this study but cite Symonds et al. 2019 – what is that reference related to?
L117: why did the cold front impact sampling? And did you mean a cold snap?
L118: did you use autosamplers? Although methods are described elsewhere, some visuals would be nice (photos/diagram of sampling strategy)
L119: what is the SENB+ pipeline? Citation?
L125: Supplemental data – over a thousand pages! I baulked when I saw that. You should include data in raw csv or txt format, i.e. not copied into a word processing document. No-one will look at that or be able to use it.
L130: if you mention software, need appropriate citation of company. Windows need OS.
L131: First mention of Ct, will lay readers (even some readers of FEMS Microbe) know what this is?
L137: 'allowances' -> 'thresholds'
L150: So LOD is at Ct = 40.
L196-197: Structures should be italicised or in inverted commas, e.g. structure 'Admin'.
L213: What R package was used?
L240: Not having flow data weakens this study. Admittedly, the approximation is reasonable in the absence of flow, but conducting both flow measurements and tracer dye studies (to generate hydrographs) would facilitate a better calculation and interpretation of the data. There are also other factors that could dramatically change the load – e.g. flow velocity and sample timing. I assume you estimated peak flow time window and sampled during that period?
L249: reference/version for package 'corrplot'
L251: Pearson assumptions – namely?
L270: 'learning' -> 'education'
L285-286: This is quite important (and preceding results) and should not be a throwaway remark. You name 'assumed' factors, but did not do any further analyses? Shifts in presentation of the virus, e.g. via a new variant, may also cause changes in viral shedding (in stool). At this point in your study, I don't think anyone had been thinking of this, but with the 'Omicron effect' clearly showing a change in the ratio of cases versus wastewater viral RNA concentration, we can suspect that variants can have a dramatic impact on how we interpret our WW data in relation to clinical observations. I wonder if you have any typing information for those presenting with the disease on campus – wild type versus alpha, delta? I know there are a few studies on this now, but not sure if there are any commentary papers published. Worth having a scan and adding something to this part of the discussion if you don't have data/capacity to investigate.
Figure 1. could you cluster your heatmap spatially rather than alphabetically? On the lower figures, why use day and not date?
L311 – its hard to understand what this mean absolute residual is – the second term is a word salad. I don't even see how this could give a coherent statistic. You are comparing daily measurements versus a reduced sampling measurement (fixed in time?). The magnitude of the measurement is of no consequence to the performance of the sampling strategy? At least not using simple comparison (X_i – Y, i = 1,…n). I would think you need a gold standard comparator (e.g. clinical) and some smoother on both frequent and less frequent sampling. Then compare the correspondence of these rolling averages. If the reduced sampling strategy was to be shown not to result in poor performance, then the loss in information should not result in a significant increase in the error with that method (could use MAE). Reading on, I think you have attempted something like that, so my first point suggests you just need to explain this method clearly – add math if need be!
L335-337: what do these 'phases' mean? Log order changes in concentration. Would load per capita be a better discretiser?
L338: again, method needs clarifying to understand what you are doing here with 'up-to-date measurement'.
L358: Surely this is obvious? Caseloads should correspond to what is measured in the wastewater + environmental noise + stochastic effects + censoring + lead/lag effects. It's those additions that lead to loss in correspondence.
L365: That is correct, no-one should be making decisions based on quantity of gene copies in wastewater (yet!), but relative changes and inflection points are very useful and are used to inform potential rising transmission (if WW is leading).

L372: You would not expect great correspondence in your estimations for the reasons you state. The magnitude of this would be testament to the performance of it, however.
L375: This was my argument to the previous comment, but I removed as it doesn't make sense, I think. You don't explicitly model this behaviour. Your estimations are based on a measured signal and an approximation of flow. Hence, the error would come in that approximation not the measured signal. However, you are quite correct with regard to decay and tracer studies with dye (for flow) and inactivated virus or a proxy (for physicochemical impacts/decay) would have given you more information to understand this.
L411: May be worth citing some normalisation literature that is out there, for the curious reader. A few papers also talk about uncertainty in wastewater surveillance for covid, that may be also useful to add to back up your caveats around weaknesses of this study (there is still a lot to do – we are not yet on top of uncertainty for WBE!).
L420: Did you also consider crAssphage?
L565: This conclusion could be stronger, just hand-waving at this point. You could elaborate on those considerations to give a more interesting wrap-up.

---What would be interesting to understand how campuses and variation in campus settings (remote, city) and their interconnectivity with wider populations are a source of viral transmission – do they act like islands when remote/low interaction, and amplifiers if situated in close proximity to the general population (i.e. seeding rate changes depending on factors related to size and distance of campuses from the major population centres?)

Author's Response to Decision Letter for (FEMSMC-2022-011)

Dear FEMS Microbes Special Collection Editors Asja Korajkic, Warish Ahmed, and Aaron Bivins and editor in chief Jana Jass,

We thank all reviewers for their thorough consideration and detailed list of suggested improvements to the clarity in the presented descriptions of the analyses, the visuals associated with the figures, and the overall language of the document. We believe by addressing each of these points thoroughly below, the final document is considerably more straightforward and easier to interpret. This response to the reviewers is also included as a separate supplemental document to preserve formatting.

Sincerely,

Cresten Mansfeldt

Assistant Professor
University of Colorado Boulder
Department of Civil, Environmental, and Architectural Engineering
Environmental Engineering Program

===

Effectiveness of building-level sewage surveillance during both community-spread and sporadic-infection phases of SARS-CoV-2 in a university campus population

Response to Reviewers

Reviewer: 1

More straight to the point sentences would make it more reader-friendly. An example; Change sentences like "The null hypothesis that there is no difference between semesters in frequency of inaccurate calls was rejected for a Fisher's exact test (p-value = 2.2x10-16)." to something short and informative like "Frequency of inaccurate calls was significantly different between semesters (Fisher's exact test, p-value = 2.2x10-16)."

To improve clarity in communication, we have edited L344 of the original text to incorporate the suggestion. Additionally, we conducted an extensive review of the language throughout the document to improve conciseness and formality.

Ending paragraphs with a one sentence conclusion would also be helpful, as right now many parts need to be read multiple times to understand what's being said instead of immediately understanding the take home message. For example: "Taken together, these data indicate that the higher caseload and resulting concentrations of SARS-CoV-2 within wastewater experienced during the Fall semester increase the absolute value of the error between the subsampled and the actual recorded daily value, but not necessarily the normalized error." So what does that mean? What is the take away exactly? This conclusion is only easy to understand when having read the paper sentence by sentence in depth, instead of it providing a short conclusion and the significance/impact of such a finding.

We agree with the reviewer that adding summary sentences at the end of paragraphs improves our manuscript. After reconsidering and restructuring the language of the results and discussion, the conclusions of each paragraph in the results and discussion stand to serve as a summary of the overall study. Line numbers reflect the revised text:

L279-281 - "Comparing these two semesters allows exploring both the utility of the data and the required resource commitment during key phases of a public health emergency."

L310-312 - "Detecting consistency in error builds confidence that the methods and approach did not bias or unevenly influence the monitored results, notably when experiencing substantially different phases in community spread."

L347-349 - "Therefore, if resources are constrained, then single-day sampling should be sufficient when considering accuracy alone."

L372-375 - "Under the conditions of our analysis, distinguishing contributions from infections in upstream structures further downstream in the pipe network during a phase of low and sporadic infection was unreliable, as demonstrated by the resulting negative population-normalized values."

L400-403 - "Overall, understanding how the sewer conveyance network itself modifies upstream detected signals as it flows downstream is important when discriminating between specific structures or watersheds, as demonstrated by the potential spatial bias detected in the Spring semester and the expanding active research in this area [Kuhn et al., 2022; Vallejo et al., 2022; Zulli et al., 2022]."

L416-418 - Supporting previous findings and applications, these PMMoV values should be considered in the future to potentially normalize the SARS-CoV-2 data [D'Aoust et al., 2021; Wolfe et al., 2022; Holm et al., 2022].

L443-446 - "Combined, these correlation analyses highlight that WBS alone does not fully recover noticeable trends in residential behavior at such a fine resolution, requiring other public health initiatives such as robust contact tracing methods to better understand the dynamics of SARS-CoV-2 concentrations in wastewater."

L452-453 - "Notably, distinct norovirus peaks were observed throughout the monitoring period (Figure 5)."

L472-474 - "This result supports daily testing when time and resources allow to ensure infrequent norovirus spikes during low overall prevalence with the potential to lead to community spread are found, corroborating the previous analysis for the SARS-CoV-2 data."

L489-492 - "This reflection of the medical findings initially supports the ability to understand community influenza burden through wastewater testing but requires further validation during a year in which influenza is at a higher prevalence."

Figures are also hard to interpret, and I am unsure I am understanding all of it. For example in figure 2; as measured values are shaded in black according to the legend, which I am not sure where I can see that (the black line?). Then it says associated lines for the reduced sampling series, but then refers to colors (which are not really lines, more shaded areas?). Not sure how informative this figure is, as to me it is more confusing than explanatory.

The reviewer raises a critical point in being able to clearly communicate the value of the Figures throughout the text. First, we have updated the legends to directly address the points raised. The as-measured values are depicted by a black line, while the reduced sampling scenarios are depicted by the shaded regions. We have updated the language accordingly on line 326 (original). We also reconsidered the shaded areas and utilized them to represent the difference between the prediction and actual value. We believe this improves the usability and connection of this figure to reflect the points raised within the text.

Figure 3 is also hard to read. What is the take home message here? Maybe just show and example from one building instead of multiple, and move the others to the supplement. That way the resolution is more clear and maybe the graphs are easier to understand. Point with arrows to areas the reader should pay attention to maybe? It is a very meaningful exploration and definitely a question in the field, the take home message is just hard to understand from looking at the figure.

We thank the reviewer for the critical feedback on how this dense Figure was received, and agree that presenting less may improve the interpretation. To improve the clarity of Figure 3, we have kept only the example from site B(A) and structure B and move the remaining to the now Supplemental Figure 7. Additionally, we have colored negative inferred values red to draw attention to the central point of this figure: that this method is not sufficient for detecting contributions from the downstream structure.

Figure 4 and 5 seem like they would be better suited for the supplemental section, with a short description in the text of the findings. These findings need additional work to really be meaningful and sound, since there are a lot of limitations and unmeasured covariates to this data, making it hard to draw conclusions from.

We agree with the reviewer that these sections were opportunities to condense the text. We moved Figure 5 to the supplemental materials and reduced the text surrounding the description of the correlation and autocorrelation. We elected to keep Figure 4 because it well summarizes a substantial number of measurements across the campaign. However, we substantially reduced the text describing the PMMoV section to now read as follows:

"Utility of Data - PMMoV Standardization. A potential approach to standardize the influence of both the sewer network and technical processing is establishing a reliable within-sample sewage indicator. During the Fall semester, F+ bacteriophage was used as a human fecal indicator, but it displayed variability over several orders of magnitude between different sample locations and days [Reeves et al., 2021]. Therefore, for the Spring semester, F+ bacteriophage was replaced by PMMoV [Rosario et al., 2009; D'Aoust et al., 2021]. Wastewater concentrations of PMMoV in this study were generally above the LOQ and displayed higher consistency than was observed with F+ bacteriophage (Figure 4). Out of 46 occasions (3% of the total 1532 measurements) in which PMMoV was found to be below the LOQ in wastewater (not including the 183 non-detects), 17 of these were from the Admin site or residential structure N. These LOQ violations likely resulted from the low and fast flow conditions at these sites [George et al., 2022]. Therefore, PMMoV generally functioned more consistently as a human fecal indicator than F+ Bacteriophage (noting, though, that they were not tested on the identical dataset). Supporting previous findings and applications, these PMMoV values should be considered in the future to potentially normalize the SARS-CoV-2 data [D'Aoust et al., 2021; Wolfe et al., 2022; Holm et al., 2022]."

Reviewer: 2

- I understand the reason for citing methods used from an earlier paper, but as a reader of papers I find it frustrating to have to go look up a different paper. It would be better to provide a precis of the method or to provide the details in the supp info

We agree with the reviewer that the addition of a summary of the previous methods would aid the readability of this manuscript. We have added the following at L103 (revised) to provide the context needed to address the material presented within this study:

"Sample Collection and SARS-CoV-2 Processing. The Fall methods and data, spanning August 25th, 2020-November 23rd, 2020, was previously presented [Reeves et al. 2021]. Briefly, 24-hr composite samples were withdrawn from 23 distinct wastewater flows using constructed autosamplers at manholes around the CU Boulder campus. Sites were labeled A-S going from the most southerly to most northerly sampled location, except for the isolation (Isolation) and administrative buildings (Admin). If specific locations received flow from multiple targeted structures, then the primary location was listed first with other contributing flows presented in brackets (e.g., A(B), E2(CBA), and G(FEDCBA)). The autosamplers withdrew approximately 10-L over 24-hr, and triplicate 50-mL subsamples were collected daily from each autosampler between 7:00 AM and 12:00 PM. After transport to the laboratory, these samples were spiked with bovine coronavirus as an internal process control, and the sample was concentrated using electronegative filter pipettes (Innovaprep, Drexel, MO) and the RNA was extracted with a commercially available kit (RNALink, Thermo Fisher, Waltham, MA), respectively. After quantifying the extracted RNA on a Qubit (Thermo Fisher), the "SENB+" pipeline was run. "SENB+" consists of a reverse-transcription quantitative polymerase chain reaction (RT-qPCR) multiplex TaqMan assay used to detect SARS-CoV-2 N (N2), SARS-CoV-2 E, bovine coronavirus, and genogroup II F+ RNA bacteriophage. All RT-qPCR analyses were performed on a QuantStudio 3 (Thermo Fisher). A daily process blank (bovine coronavirus spiked ultrapure water) was run with the wastewater samples, and at least three no-template controls were used on the RT-qPCR plate."

- It is not completely clear to me what QC was done, especially to control for lab contamination. This can be especially important in a low prevalence context when most samples are negative / low positive and only one or a few samples are strong positives. Were extraction sample blanks / extraction blanks run, if yes, what frequency?

This was a critical oversight in communication, and we have addressed this concern specifically within the summary attached to the previous question. A process blank was run daily, and at least three technical qPCR blanks were run.

-in terms of assay performance, how did the two SARS assays compare in terms of virus numbers detected (E vs N)?

We thank the reviewer for raising this key quality check. We believe that it is addressed on L125 (original), in which we report a Pearson's r value between E and N of 0.93, and in Supplemental Figure 3.

- have other fecal indicators been considered? While PMMoV is a fecal indicator, I am not sure how useful it is as a universal marker for normalizing sewage strength given that it is derived from diet, rather than being part of the human gut flora. While FRNA meet this criterion, they are highly variable. Data I have seen suggests fecal indicators such as Bacteroides are more stable and also at higher numbers compared with FRNA

Other fecal indicators were considered in preliminary analysis, but the PMMoV was ultimately selected as a wider number of WBS applications have adopted this as a normalizing control [e.g., D'Aoust et al., 2021; Wolfe et al., 2022] and in comparison have found it to exceed RNaseP in stability and perform similar to crAssphage [Holm et al., 2022]. An additional benefit of selecting PMMoV over Bacteroides is that PMMoV is detectable utilizing the same extraction kit and qPCR run, without having to apply a separate DNA recovery.

D'Aoust, P.M., Mercier, E., Montpetit, D., Jia, J.-J., Alexandrov, I., Neault, N., Baig, A.T., Mayne, J., Zhang, X., Alain, T., Langlois, M.-A., Servos, M.R., MacKenzie, M., Figeys, D., MacKenzie, A.E., Graber, T.E., Delatolla, R., 2021. Quantitative analysis of SARS-CoV-2 RNA from wastewater solids in communities with low COVID-19 incidence and prevalence. Water Res. 188, 116560. https://doi.org/10.1016/j.watres.2020.116560

Wolfe, M.K., Duong, D., Bakker, K.M., Ammerman, M., Mortenson, L., Hughes, B., Martin, E.T., White, B.J., Boehm, A.B., Wigginton, K.R., 2022. Wastewater-based detection of an influenza outbreak. Preprint. medRxiv. https://doi.org/10.1101/2022.02.15.22271027

Holm, R.H., Nagarkar, M., Yeager, R.A., Talley, D., Chaney, A.C., Rai, J.P., Mukherjee, A., Rai, S.N., Bhatnagar, A., Smith, T., 2022. Surveillance of RNase P, PMMoV, and CrAssphage in wastewater as indicators of human fecal concentration across urban sewer neighborhoods, Kentucky. FEMS Microbes, 3, xtac003, https://doi.org/10.1093/femsmc/xtac003

- I am not sure if the authors have considered the variability in virus excretion between COVID cases. Depending on the review, something like only 40-50% of cases excrete SARS-CoV-2 in feces, so this could also explain variability in virus numbers when examining virus detection from individual locations in series (A, B, C etc)

The reviewer is correct in that we did not consider the probability that an individual would actually contribute virus to the system, and additionally we also ignored the variable defecation patterns of individuals (multiple times per day to a few times per week). The main takeaway point was not contingent on the variability of the contribution, but rather the variability of within sewer processes. While the variability in viral shedding certainly affects our measurements as they compare to case numbers overall, this would result in the negative values which were observed in our pipeline dilution analysis. Because these samples were collected as 24-hour composites, the upstream and downstream autosamplers were drawing from the same flow contributed by the upstream structure. Therefore, the detection upstream is indicative that an individual is indeed infected, and this signal should be conserved based on mass flow properties on the downstream signal. However, multiple potential confounding factors such as feces transporting slower than water at these upstream sites, the presence of toilet-paper rafts and oversampling effects, and other within sewer processes likely leads to these variations. The variability in the contribution would affect the analysis of the linkage to medical services data; however, this comparison not expounded on in great depth.

- Have the authors considered if different strains result in different virus excretion patterns? The data from SARS WBE that I have been involved in suggests that delta (probably also omicron) are shed at levels orders of magnitude higher than the earlier variants

We did not pursue this exploration within the current manuscript as the time period covers primarily the original and alpha-strain of SARS-CoV-2, suspending the campaign prior to the emergence and proliferation of the delta strain in the summer of 2021.

- Figure 2 mentions black shading, do you mean the black line in the graph?

The reviewer is correct and this has been now corrected, please refer to our comment to reviewer 1.

Reviewer: 3

Comments to the Author

In summary, the following areas need improvement:
- Clarity, especially in description of methods (particularly stats/numeric/relational) and when making overarching statements (of purported fact)

We thank the reviewer for highlighting this need, and the methods were holistically revised to more clearly and simply communicate the activities of this study. Although the list of edits was too extensive to detail here, a concrete example of the revamped approach is converting the confusing language surrounding the flowpath into equations:
"The daily viral loads contributed by only B, E, and F and D (to be referred to together as FD) was calculated by subtracting known upstream signals from the known, combined, downstream signals:
PopB * [SARS-CoV-2]B = PopB(A) * [SARS-CoV-2]B(A) - PopA * [SARS-CoV-2]A Eq. 2
PopE * [SARS-CoV-2]E = PopE(CBA) * [SARS-CoV-2]E(CBA) - PopB(A) * [SARS-CoV-2]B(A) – PopC * [SARS-CoV-2]C Eq. 3

PopFD * [SARS-CoV-2]FD = PopG(FEDCBA) * [SARS-CoV-2]G(FEDCBA) - PopE(BCA) * [SARS-CoV-2]E(BCA) - PopE2 * [SARS-CoV-2]E2 Eq. 4
These estimations were then compared to the Spring medical services data for B, E, and FD to explore accuracy."
As per the suggestion of another reviewer, substantial areas of speculation around the correlation analyses and the PMMoV discussion were removed, shortening the length of the document as well. Reconsidering the text overall, elements of the materials and methods were also removed from the results and discussion, overall leading to a shorter and cleaner document.
- The style of writing is generally good, but does become informal in places. I did not point these out, but would recommend having an independent and qualified party review the language in the manuscript.

We thank the reviewer for highlighting this need. The document was reconsidered to improve the formality of the language. After revision, we noted that informality often correlated with the switch into first-person text, therefore we focused on removing that element from the document. Additionally, adverb and adjective usage was reconsidered throughout to improve on the overall formality as well as clarity.

- The supplementary material, i.e. the data, is best delivered as data files rather than copied into unusuable format (i.e. text processing document).

We agree that the format the data was delivered in is unusable and are actively working with the editors to ensure that the as-submitted txt and xls files remain in machine readable format. Unfortunately, the portal converted our data into this format upon submission for the pdf version of the manuscript. The html version preserved the readability of the material, and we will supply our data files in a conventional format for this submission.

The manuscript touches on a lot of questions that are under discussion related to the value of WBE for public health - it is good that you comment on some of the uncertainty, and complexities, in your study and utility of WBE generally. I think the manuscript is dense with information, mostly valuable, if not entirely unique (many similar studies out there, but this at least approaches from a fairly novel angle, if not always successfully). I would consider publication after major revision.

Thank you for this summary of our work, and we share the view that the originality of the work resides in the contextualization and exploration of resource commitment. With WBS campaigns adopted across the country, this novel exploration will be able to apply refreshed analysis of the shared information between these studies. Additionally, as per multiple valuable comments, we have revised the manuscript to assist in the readability and accessibility of the presented density of information.

L43: "scale with the objectives" -> should be scale with the importance or level of risk/impact to public health – or just 'level of surveillance is objective dependent'? Objectives are not quantifiable in the general sense.

We have modified this sentence to be more intelligible, by changing the phrase to, "scale to meet the objectives of the monitoring campaign" at L43 (original)

L48-50: This depends. Wastewater monitoring specifically to inform public health action has precedents, but it has not been 'widely applied'. While you could argue that WBE during COVID has seen wider application, it is certainly not standardised where it is implemented. This forms one of the fundamental 'next steps' for WBE in my opinion – this includes the end to end SOPs/protocols/processes.

This introductory section has been revised for simplicity and directness to read as follows:

"Wastewater-based surveillance (WBS) has been successfully applied to monitor entire sewage-drainage catchments to individual buildings as a public health response to the coronavirus disease (COVID-19) pandemic [Keshaviah et al., 2021; Kirby et al., 2021; McClary-Gutierrez et al., 2021; Naughton et al., 2021]. The second emergent severe acute respiratory syndrome coronavirus (SARS-CoV-2) which causes COVID-19 is responsible for an estimated 594,000,000 infections and 6,446,000 deaths globally as of August 22, 2022 [WHO 2019; Peccia et al., 2020, Spurbeck et al., 2021, Medema et al., 2020].

Globally, select college campuses adopted WBS to monitor on-campus residences and complement individualized medical and screening programs"

L59: At least one of these references (Sweetapple et al) is non-US campus WBE. Please rephrase, e.g. "Across the United States and elsewhere" (L55). Do you have data on number of campuses in total (across US at least), and possible population covered?

We thank the reviewer for highlighting this error. We have adjusted the language on L55 (original) to "Globally".

On L60, we have also included the following:
"As of August 22, 2022, 282 universities are publicly reported to have run WBS campaigns targeting SARS-CoV-2 [Naughton et al., 2021]. However. the total population covered by these campaigns remains ambiguous resulting from the combined factors of incomplete or transient monitoring of those on campuses (e.g., inability to monitor specific locations, commuters, employees)."

L70: Not sure 'robust' is the correct word here. 'significant/contiguous' ?

In rereading the introduction, this entire section was removed for easier readability and the key information is presented in lines 88-99 of the revised text. Notably, this will just be stated as "community spread" in the remainder of the document.

L76-78: I am not convinced this provides the insight you suggest. It provides knowledge of local scale utility of WBE, I'm not sure how it provides national indication of progression. Please elaborate.

In rereading the introduction, this entire section was removed for easier readability and the key information is presented in lines 88-99 of the revised text.

L81: do you mean 'social network', could be rephrased as looks like a missing word after 'social' – societal behaviours?

We have rephrased this section to read as follows on l78 of the revised text:
"Alterations in social activity also influence the prevalence of SARS-CoV-2, and exploring the dynamics captured by WBS data or overlaying the results onto other human behavior datasets may inform the spatial and/or temporal resource commitment required to monitor a given community effectively"

L86: Font size changes

We have standardized the font size to 11.

L83-88: A bit confusing. You seem to frame the argument around 'individualized testing' but do not mention how this factors into your sewer sampling strategy. Or does it not matter?

We agree with the reviewer that this was phrased in a confusing way. This was meant as a general statement about the utility of fine-scale wastewater surveillance—that individualized testing could be deployed in response to the results of wastewater surveillance to rapidly identify individuals—rather than being a description of the methods used for this study. We have rephrased as follows on L68 of the revised text:

"A main intent of monitoring smaller sewer catchments or at the building-level is being able to deploy additional individualized testing in response to results from wastewater, identify infected individuals rapidly, and thus medically intervene early to prevent severe disease and further viral spread".

L94-95: This is a spurious statement. The article you cite [Xie et al] discusses chemical marker usage and its impact on WBE data (i.e. for normalization), but your statement implied the more complex social interactions and behaviours have been researched and I would expect to see some social science/epidemiology papers discussing this. Although I am aware of some studies, I don't think the true impact of mobility, demographics and bathroom habits have been studied systematically – some work on

bathroom habits on air transport/terminals has been performed [in preparation, not published], but at larger scale, I'm not sure. Please elaborate or temper the statement.

We thank the reviewer for highlighting this reference error. The Xie et al., 2022 paper was to appear in the list before dicussing process normalization. The Wright et al., 2022 paper, discussing the overlay of WiFi data onto wastewater was intended to be cited here. This section now reads as follows [L78-81]:

"Alterations in social activity also influence the prevalence of SARS-CoV-2, and exploring the dynamics captured by WBS data or overlaying the results onto other human behavior datasets may inform the spatial and/or temporal resource commitment required to monitor a given community effectively [Wright et al., 2022]."

L98: why is it key? What is the motivator for WBE when flue and norovirus surveillance clinically appears to be generally effective…what about enterovirus, polio – which have precedent, but are still of concern [given recent news].

Enterovirus and polioviruses are indeed of concern, but were not selected as the initial exploration of alternate monitored viral particles. The intent of this selection was to more broadly show that the resource commitment shifts for each virus. Therefore, the original sentence was revised to be in more inline with the intent of the study (L82-85 revised):

"Prior to deploying WBS for these new targets, each virus requires additional validation and optimization. For example, norovirus is a commonly identified pathogen shed within feces whereas influenza prevalence has been previously shown to be disconnected from wastewater concentrations, both providing unique considerations for WBS."

L109: you discuss this study but cite Symonds et al. 2019 – what is that reference related to?

This statement was intended to demonstrate that PMMoV has previously been used as a fecal indicator. To clarify the meaning behind this citation, we have moved the reference to the materials and methods.

L117: why did the cold front impact sampling? And did you mean a cold snap?

We thank the reviewer for this correction, as cold snap is more accurate to describe our meaning than cold front. The cold snap interrupted sampling, because collected wastewater would freeze in the inlet line and sampling port. This is now summarized at L123-125 (revised) as follows:

"Sampling was suspended February 11th-18th because of an extended period of cold temperatures freezing the inlets to sample stations and causing hazardous exposure conditions to the sample collectors."

L118: did you use autosamplers? Although methods are described elsewhere, some visuals would be nice (photos/diagram of sampling strategy)

Autosamplers were used for this campaign. We have added a summary of our methods in response to a previous suggestion which describes our sampling strategy. We elected to not include imagery to save on space.

L119: what is the SENB+ pipeline? Citation?

The SENB+ pipeline is now described in the summary of our methods.

L125: Supplemental data – over a thousand pages! I baulked when I saw that. You should include data in raw csv or txt format, i.e. not copied into a word processing document. No-one will look at that or be able to use it.

Thank you for raising this point; we share in this concern resulting from the submission process. As addressed in an earlier point, the HTML version of the document appears as intended, but the PDF

translates all supplied material rather than linking. This will additionally be reiterated in the cover letter and insured is uploaded properly on final submission.

L130: if you mention software, need appropriate citation of company. Windows need OS.

We have included citations for QuantStudio 3 and 5 in line 130 (original), as well as for R in line 135 (original).

L131: First mention of Ct, will lay readers (even some readers of FEMS Microbe) know what this is?

We have now defined this as "cycle threshold".

L137: 'allowances' -> 'thresholds'

We have changed allowances to thresholds at L137 (original).

L150: So LOD is at Ct = 40.

We thank the reviewer for this clarification. While the LoD has a maximum possible value of 40, the LoD was calculated on a plate-by-plate basis, to be below amplifications in the negative control reactions. We have corrected the error in L149-153 (revised) from "above" to "below". This section now reads as follows:

"The lowest-concentration standard level remaining after cleaning for each plate and target established the limit of quantification (LoQ). The limit of detection (LoD) was set at the maximum cycle (the 40th cycle) or lower if non-specific amplification was observed in the negative control reactions. The final processed standard curves were generated for each plate and every target and used in quantification.

L196-197: Structures should be italicised or in inverted commas, e.g. structure 'Admin'.

We have italicized the structures and stations mentioned within the text of our manuscript.

L213: What R package was used?

The R package rstatix (version 0.7.0) was used for this analysis. We have incorporated this into L212 (original).

L240: Not having flow data weakens this study. Admittedly, the approximation is reasonable in the absence of flow, but conducting both flow measurements and tracer dye studies (to generate hydrographs) would facilitate a better calculation and interpretation of the data. There are also other factors that could dramatically change the load – e.g. flow velocity and sample timing. I assume you estimated peak flow time window and sampled during that period?

We agree with the reviewer that flow data would likely minimize much of the variability observed in this analysis. These samples were collected as a 24-hour composite, which we have now clarified in the summary of our methods.

L249: reference/version for package 'corrplot'

Version 0.92 of corrplot was used for this analysis, which we have indicated at line 248. And a citation has been added to L249 (original).

L251: Pearson assumptions – namely?

Failed assumptions included the presence of outliers within the data, as well as the assumption of normality which is required for significance testing of the Pearson correlation coefficient. We have revised this section to read as follows (L246 revised):

“Spearman's correlation was used to avoid Pearson correlation assumptions of normality and lack of outliers”

L270: ‘learning’ -> ‘education’

We have changed learning to education in L270 (original).

L285-286: This is quite important (and preceding results) and should not be a throwaway remark. You name ‘assumed’ factors, but did not do any further analyses? Shifts in presentation of the virus, e.g. via a new variant, may also cause changes in viral shedding (in stool). At this point in your study, I don’t think anyone had been thinking of this, but with the ‘Omicron effect’ clearly showing a change in the ratio of cases versus wastewater viral RNA concentration, we can suspect that variants can have a dramatic impact on how we interpret our WW data in relation to clinical observations. I wonder if you have any typing information for those presenting with the disease on campus – wild type versus alpha, delta? I know there are a few studies on this now, but not sure if there are any commentary papers published. Worth having a scan and adding something to this part of the discussion if you don’t have data/capacity to investigate.

We agree that the presentation of unique symptoms for each variant greatly influences that contribution to and recovery of SARS-CoV-2 from wastewater. Over this time period, only the wildtype and alpha were detected. This time window likely had less to do with the emergence and circulation of a new variant, but the resumption of social activities and human contact. Additionally, the full isolation/contact tracing was operating efficiently at this point, essentially excluding more rapidly from being detected throughout the sewer network. To clarify the intent of this section, without speculating on areas in which valuable data is now being released from other sources, we modified the sentence to read as follows:

“Speculatively, detection, contact tracing, and isolation (i.e., removal from detection through wastewater) became more routine in the Spring, vaccines became more available toward the conclusion of the sampling campaign, and human behavior and on-campus occupancy shifted, all likely contributing to this small disconnect [Bivins and Bibby, 2021]”

Figure 1. could you cluster your heatmap spatially rather than alphabetically? On the lower figures, why use day and not date?

To clarify this within the text, we have added the following line to L292-294 of the revised manuscript.
“The alphabetical designation is arranged spatially, with A being the furthest South and S being the furthest North sample station on the sewage network.”
Day is used rather than date as two separate time periods are compared on the same graph, and this additionally assist anonymity of the presented case data.

L311 – its hard to understand what this mean absolute residual is – the second term is a word salad. I don’t even see how this could give a coherent statistic. You are comparing daily measurements versus a reduced sampling measurement (fixed in time?). The magnitude of the measurement is of no consequence to the performance of the sampling strategy? At least not using simple comparison (X_i – Y, i = 1,…n). I would think you need a gold standard comparator (e.g. clinical) and some smoother on both frequent and less frequent sampling. Then compare the correspondence of these rolling averages. If the reduced sampling strategy was to be shown not to result in poor performance, then the loss in information should not result in a significant increase in the error with that method (could use MAE). Reading on, I think you have attempted something like that, so my first point suggests you just need to explain this method clearly – add math if need be!

To assist in the clarification of the point, we altered the methods to read as follows:

“Decreased Sampling Frequency Exploration. To compare the accuracy of daily to less frequent sampling scenarios in both times of high community spread (Fall) and times of low/sporadic community spread (Spring), the Fall and Spring data were subsampled by weekday according to the following sampling schemes: Monday, Wednesday, Friday (MWF); Monday only (M), and Saturday only (S). The reduced sampling scheme excluded actual measured values (e.g., MWF excluded Tuesday, Thursday, Saturday,

and Sunday samples) and replaced those excluded values with the most previous measurement (e.g., Tuesday values were replaced by Monday values). For each of these schemes, differences between the replaced value and the value that it replaced were calculated daily for each sampling site. For each residential structure, the differences from each scheme were summed for the entire semester and normalized over the semester-total copies per liter wastewater for that structure. A pairwise Wilcoxon test was then implemented in R using the rstatix (version 0.7.0) package to compare the ratios for all residential structures between the Fall and Spring semester, grouped by the subsampling schedule [Kasambra 2020].

And the results to read as follows:

"Temporal Resource Commitment - Effect of Decreased Sampling Frequency. Across all building-level campus WBS campaigns, sampling frequencies have ranged from weekly to daily [Betancourt et al., 2021; Gibas et al., 2021; Karthikeyan et al., 2021; Scott et al., 2021]. Less frequent sampling schedules may be necessitated by the financial and labor cost associated with daily sample collection, processing, and analysis. Whether reduced sampling schedules are informative during times of sporadic infection/low community spread (e.g., Spring) or times of high community spread (e.g., Fall) remains less explored (Figure 2). Therefore, subsampling the daily nature of the reported campaign into artificial MWF, M, and S campaigns enables exploring the effort-to-accuracy required. For all subsampling scenarios, the mean difference between the concentration value that was excluded, and the value used to replace was higher in the Fall semester than in the Spring (Wilcoxon rank order test supported unequal means). When these differences were normalized over the concentration measurements that were excluded, the two semesters were indistinguishable (Wilcoxon rank order test support of equal means). Taken together, the higher caseload and resulting concentrations of SARS-CoV-2 within wastewater during the Fall semester resulted in a higher absolute difference between the replaced and the actual recorded daily value, but not necessarily the normalized, when compared to the Spring. Detecting consistency in the normalized difference builds confidence that the methods and approach did not bias or unevenly influence the monitored results, notably when experiencing substantially different phases in community spread."

L335-337: what do these 'phases' mean? Log order changes in concentration. Would load per capita be a better discretiser?

For larger sewer networks, load per capita likely provides an ample opportunity to provide a consistent discretizer. However, within this monitoring project, our sites ranged from approximately 70-7000 with a mean of approximately 200 individuals monitored per site, introducing substantial variation with population misestimates or fluctuations (as indicated by the further pipeflow analysis). In reconsidering the entire narrative, we realized we were employing phase in multiple contexts, leading to further confusion. Rather than keeping this as "phase", we replaced this labeling scheme simply as "partition". We believe this assists the flow of the document.

L338: again, method needs clarifying to understand what you are doing here with 'up-to-date measurement'.

We removed a substantial amount of the jargon in this section. This sentence now reads as follows:

"This process enabled calculating the number of replaced-value days (i.e., days artificially substituted by the previous measurement) in each scenario that correctly partitioned with the excluded sample (Figure 2.B)."

L358: Surely this is obvious? Caseloads should correspond to what is measured in the wastewater + environmental noise + stochastic effects + censoring + lead/lag effects. It's those additions that lead to loss in correspondence.

Agreed! We were intending a different discussion surrounding the need for daily sampling that was succinctly captured in a sentence within the previous paragraph. In reconsideration of this section, and the confusing nature of the presentation, we opted to remove to save on space and end this analysis with a concrete final statement as follows:

“Therefore, if resources are constrained, then single-day sampling should be sufficient when considering accuracy alone.”

L365: That is correct, no-one should be making decisions based on quantity of gene copies in wastewater (yet!), but relative changes and inflection points are very useful and are used to inform potential rising transmission (if WW is leading).

We definitely agree on this point! But to save on speculative statements (as highlighted as an area of improvement above) we opted to remove this section and conclude as mentioned in the point above.

L372: You would not expect great correspondence in your estimations for the reasons you state. The magnitude of this would be testament to the performance of it, however.

This is addressed with point L375 below.

L375: This was my argument to the previous comment, but I removed as it doesn’t make sense, I think. You don’t explicitly model this behaviour. Your estimations are based on a measured signal and an approximation of flow. Hence, the error would come in that approximation not the measured signal. However, you are quite correct with regard to decay and tracer studies with dye (for flow) and inactivated virus or a proxy (for physicochemical impacts/decay) would have given you more information to understand this.

Potentially, however, flow is not the only factor to consider. Within sewer processes and human behavior complicate the direct one-to-one relationship as mentioned with the proxy or dye tracer. For example, visually at many of our sites, the fecal matter did not necessarily flow at the same rate as the water because of either “toilet-paper rafts” forming or the water level was low enough that feces would be immobilized by the friction of the pipe itself. Therefore, the variability is a combination of both flow misestimation and the inherent fingerprint of a low-flow gravity fed system. As noted, the final signal detected through the qPCR assay is likely the lowest origin of variability. Therefore, as the reviewer suggests, future WBS campaigns should invest more into exploring the behavior of the network itself through dye tracer studies in both the liquid and solid phases and decay experiments.

L411: May be worth citing some normalisation literature that is out there, for the curious reader. A few papers also talk about uncertainty in wastewater surveillance for covid, that may be also useful to add to back up your caveats around weaknesses of this study (there is still a lot to do – we are not yet on top of uncertainty for WBE!).

We agree with the reviewer that this section would be improved with the addition of literature. We corrected the Wolfe citation tp refer to Wolfe et al., 2021 and have additionally cited the more comprehensive Holm et al., 2022.

Wolfe, M.K., Archana, A., Catoe, D., Coffman, M.M., Dorevich, S., Graham, K.E., Kim, S., Grijalva, L.M., Roldan-Hernandez, L., Silverman, A.I. and Sinnott-Armstrong, N., 2021. Scaling of SARS-CoV-2 RNA in settled solids from multiple wastewater treatment plants to compare incidence rates of laboratory-confirmed COVID-19 in their sewersheds. Envir. Sci. & Tech. Letters, 8(5), pp.398-404. https://doi.org/10.1021/acs.estlett.1c00184

Holm, R.H., Nagarkar, M., Yeager, R.A., Talley, D., Chaney, A.C., Rai, J.P., Mukherjee, A., Rai, S.N., Bhatnagar, A., Smith, T., 2022. Surveillance of RNase P, PMMoV, and CrAssphage in wastewater as indicators of human fecal concentration across urban sewer neighborhoods, Kentucky. FEMS Microbes, 3, xtac003, https://doi.org/10.1093/femsmc/xtac003

L420: Did you also consider crAssphage?

We did not consider crAssphage within the current study. Although crAssphage has been demonstrated as a successful within-wastewater normalization target, it also has been shown to perform similarly to PMMoV [Holm et al., 2022], the design of our quad-plex qPCR assay necessitated that we select only a

single target, and at the time of selection, PMMoV was more widely applied and accepted as a normalization control.

L565: This conclusion could be stronger, just hand-waving at this point. You could elaborate on those considerations to give a more interesting wrap-up.

We streamlined the statement and rewrote the structure to be less of a bulleted list and a more direct description of the need for understanding the temporal and spatial resources required to operate an effective campaign (L496 revised):

"Linking the resource requirements for effective WBS to the prevalence of the virus within a community informs operators on required sampling frequency and the effort overall required to maintain effective campaigns. For example, monitoring during low-viral prevalence (i.e., Spring 2021) introduced unique resource constraints. Although overall caseloads were lower in the Spring semester, WBS was still successfully implemented to capture individual cases of COVID-19 and monitor overall community transmission of SARS-CoV-2. Additionally, the effect of simulated decreased temporal sampling resolution during times of the lower overall transmission in Spring 2021 was found to display lower error in estimating the daily copies per liter wastewater when compared to times of high transmission in Fall 2020. However, Spring additionally displayed a lower overall accuracy at tracking the relevant stage of community transmission within the individual structures. Therefore, an inverse resource commitment is required, with a community experiencing a lower SARS-CoV-2 prevalence requiring a more frequent sampling schedule to ensure the dynamics of the virus are accurately represented during the crucial introduction and initial spread phases. When considering the spatial deployment of resources, error was also noted in attempting to infer the viral load contribution from a single residential structure in a wastewater stream with mixed inputs in the spatial analysis, with this approach being deemed ineffective because several days were calculated to have a negative viral load in downstream structures. Therefore, samples should be withdrawn as near to the monitored subsection of the community as possible, because combined flows may introduce errors in interpretation. However, even with a near building-level resolution across a university campus, correlations between residential structures were nearer to zero overall for the Spring semester when compared to the Fall semester. This indicated that although correlations were relatively strong when high-prevalence of SARS-CoV-2 was detectable across campus, the social factors behind the spread of the virus during low prevalence could not be explained by relationships in wastewater concentrations alone. Several of these WBS challenges associated with low-prevalence were also noted for norovirus and influenza, although the data for these viruses largely reflected the low-community spread noted overall by the CDPHE and CDC data. As WBS application expand effectively monitor new targets, continuing to understand and optimize the spatial and temporal resource commitment required to achieve data supportive of specific interventions of viral spread within a population ensures the maturation of WBS into a cost-effective, widely accessible, and adaptable technique."

---What would be interesting to understand how campuses and variation in campus settings (remote, city) and their interconnectivity with wider populations are a source of viral transmission – do they act like islands when remote/low interaction, and amplifiers if situated in close proximity to the general population (i.e. seeding rate changes depending on factors related to size and distance of campuses from the major population centres?)

This is a great analysis to perform, but falls outside the scope of the current study and would require a separate focused analysis to provide in depth and clear results. This would be a great metaanalysis connecting in the efforts of studies across the United States or globally, an effort likely already underway by efforts such as the National Wastewater Surveillance System at the Centers of Disease Control.

<u>Reviews of manuscript FEMSMC-2022-011.r1</u>

Reviewer 1: Dr Merel Bot, UCLA
Recommendation: Accept

Such an improvement! Very nice to read. The text is a lot more clear and concise, and the figures easier to interpret. The summary sentences at the end of the paragraphs are very helpful and well written. The data and conclusions drawn from it are very meaningful to the field.
